# Social Mobilization and Community Engagement Central to the Ebola Response in West Africa: Lessons for Future Public Health Emergencies

**DOI:** 10.9745/GHSP-D-16-00226

**Published:** 2016-12-23

**Authors:** Amaya M Gillespie, Rafael Obregon, Rania El Asawi, Catherine Richey, Erma Manoncourt, Kshiitij Joshi, Savita Naqvi, Ade Pouye, Naqibullah Safi, Ketan Chitnis, Sabeeha Quereshi

**Affiliations:** a United Nations Mission for Ebola Emergency Response (UNMEER), New York, NY, USA.; b United Nations Children's Fund (UNICEF), New York, NY, USA.; c UNICEF, Monrovia, Liberia.; d UNICEF, Freetown, Sierra Leone.; e UNICEF, Regional Office, Dakar, Senegal.; f UNICEF, Conakry, Guinea.

## Abstract

Following the World Health Organization (WHO) declaration of a Public Health Emergency of International Concern regarding the Ebola outbreak in West Africa in July 2014, UNICEF was asked to co-lead, in coordination with WHO and the ministries of health of affected countries, the communication and social mobilization component—which UNICEF refers to as communication for development (C4D)—of the Ebola response. For the first time in an emergency setting, C4D was formally incorporated into each country's national response, alongside more typical components such as supplies and logistics, surveillance, and clinical care. This article describes the lessons learned about social mobilization and community engagement in the emergency response to the Ebola outbreak, with a particular focus on UNICEF's C4D work in Guinea, Liberia, and Sierra Leone. The lessons emerged through an assessment conducted by UNICEF using 4 methods: a literature review of key documents, meeting reports, and other articles; structured discussions conducted in June 2015 and October 2015 with UNICEF and civil society experts; an electronic survey, launched in October and November 2015, with staff from government, the UN, or any partner organization who worked on Ebola (N = 53); and key informant interviews (N = 5). After triangulating the findings from all data sources, we distilled lessons under 7 major domains: (1) strategy and decentralization: develop a comprehensive C4D strategy with communities at the center and decentralized programming to facilitate flexibility and adaptation to the local context; (2) coordination: establish C4D leadership with the necessary authority to coordinate between partners and enforce use of standard operating procedures as a central coordination and quality assurance tool; (3) entering and engaging communities: invest in key communication channels (such as radio) and trusted local community members; (4) messaging: adapt messages and strategies continually as patterns of the epidemic change over time; (5) partnerships: invest in strategic partnerships with community, religious leaders, journalists, radio stations, and partner organizations; (6) capacity building: support a network of local and international professionals with capacity for C4D who can be deployed rapidly; (7) data and performance monitoring: establish clear C4D process and impact indicators and strive for real-time data analysis and rapid feedback to communities and authorities to inform decision making. Ultimately, communication, community engagement, and social mobilization need to be formally placed within the global humanitarian response architecture with proper funding to effectively support future public health emergencies, which are as much a social as a health phenomenon.

## Abstract (precis)

Key lessons for the crucial components of social mobilization and community engagement *in this context*:

- Invest in *trusted local community members* to facilitate community entrance and engagement.
- Use key communication *networks and channels with wide reach* and relevance to the community, such as radio in low-resource settings or faith-based organizations.
- Invest in *strategic partnerships* to tap relevant capacities and resources.
- Support a *network of communication professionals* who can deploy rapidly for lengthy periods.
- Balance *centralized mechanisms* to promote consistency and quality with *decentralized programming* for flexibility and adaptation to local needs.
- *Evolve communication approaches and messaging* over time with the changing outbreak patterns, e.g., from halting disease transmission to integration and support of survivors.
- Establish clear *communication indicators* and analyze and share data in real time.

## INTRODUCTION

In December 2013, an outbreak of Ebola Virus Disease (EVD) began in West Africa, spreading through Guinea, Liberia, and Sierra Leone. In July 2014, the World Health Organization (WHO) declared the outbreak a “Public Health Emergency of International Concern.”^1^ By March 2016, when the Emergency Committee on Ebola convened by WHO concluded that the outbreak no longer constituted a public health emergency,^2^ a total of 28,616 confirmed, probable, and suspected cases had been reported, more than 11,310 people had died, and 23,588 children had lost one or both parents or their primary caregiver.^3^^,^^4^ Although the region is now considered mostly Ebola-free, there is a general recognition that Ebola or other emerging public health issues will continue to pose a threat, highlighting the need for continued vigilance and preparedness.

Considered the most severe in the history of the disease, the 2013–2016 Ebola outbreak affected some of the world's most vulnerable communities and countries recovering from years of destructive civil war and unrest. An initial underestimation of the scope of the outbreak contributed to delays in funding, which in turn contributed to a slow start to the response.

Once the response hit the ground, it was initially focused on containing EVD and establishing the supply-side pillars related to surveillance, logistics, and, in particular, burials. Communities had been taking action to manage their own risks, many of which paid dividends,^5^ but the formal response at that time paid little attention to working within community structures and did not acknowledge traditional community coping strategies and influences on behavior. Rumors and misconceptions circulated widely because community members mistrusted messaging from formal communication channels. These poor community linkages and poor quality of services as a result undermined community confidence, effective social mobilization, and ultimately the response itself.

As the outbreak progressed beyond initial projections, and given the limitations of clinical approaches and weak local systems, pressure increased for community engagement and social mobilization to be central to changing behavior to prevent and control the outbreak.^6^ According to one evaluation of this component of the global response^7^:

The predominance of top-down communication in the early stage of the response reflects the way the Ebola response initially sidelined community engagement as a critical operational tool. Early Ebola messaging and response strategies were symptomatic of this, and too often failed to meet the needs and realities confronting affected populations.

For the first time in emergency contexts, social mobilization and community engagement was included as a “cluster system” (also known as a “pillar”) in the 3 most affected countries, representing a key area of focus for the response. These cluster systems were led by the ministries of health and their corresponding technical units with support from United Nations (UN) agencies and civil society organizations. Although variations existed among the 3 countries, the other pillars commonly included media and communication, epidemiology/surveillance, case management/contact tracing, infection control, laboratories, burials, logistics/supplies, psychosocial support and child protection, and other sectors such as water and sanitation, HIV/AIDS, health, nutrition, and education.

The main function of the social mobilization and community engagement pillar was to coordinate efforts and design a strategy to focus on key behaviors, including measuring and reporting on key performance indicators. The United Nations Children's Fund (UNICEF) was designated as co-lead for this pillar with government and civil society counterparts in each of the countries, while working closely with many other partners.

Various terminology is used to describe working with communities to achieve individual and/or collective change. The countries affected by Ebola used the terms social mobilization and community engagement almost interchangeably, in addition to the term communication for development (C4D). UNICEF uses the term C4D to encompass both social mobilization and community engagement. As such, C4D is a 2-way process for sharing ideas and knowledge, including social norms, using a range of communication tools and other approaches that empower individuals and communities to change behavior and take actions to improve their lives. In an emergency, C4D can help facilitate change at multiple levels—from leveraging support to influence and implement policies, to motivating and mobilizing civil society, to actively empowering households and communities to identify problems, propose solutions, and act upon them.^8^

This article describes the lessons learned in social mobilization and community engagement in the context of the emergency response to the Ebola outbreak. These lessons draw primarily on an analysis of UNICEF's C4D work in the 3 affected countries, but they also build on and are complemented by lessons and assessments conducted by other partners involved in the Ebola response.

## METHODOLOGY

The purpose of this assessment was to identify lessons learned from the Ebola response in West Africa, with a particular focus on C4D, using a mix of the following 4 methods:

**Literature review:** We began with a desk review of key independent and interagency documents from UNICEF and partner agencies, such as the UN Mission for the Ebola Emergency Response (UNMEER), WHO, the UN Office for the Coordination of Humanitarian Affairs (OCHA), Health Policy Group, Oxfam, Médicins San Frontières, and Catholic Relief Services. We also drew useful insights from key meeting reports, such as the UNMEER/UNICEF regional consultation in Freetown, Sierra Leone, in March 2015 and the interagency meeting hosted by Oxfam in September 2015, which included a wide range of NGO partners. Finally, we conducted a wider online search of relevant peer reviewed articles and grey literature published between December 2013 and March 2016 focused primarily on lessons learned, community engagement, and communication and social mobilization in the Ebola response, and analyzed available Standard Operating Procedures (SOPs) on Ebola, with a specific focus on C4D (including social mobilization and/or community engagement).

**Structured expert discussions:** Based on the literature review and the analysis of SOPs, we conducted a structured discussion in June 2015 with more than 90 UNICEF and civil society participants across West and Central Africa to elicit key lessons learned. To further explore the lessons identified by this initial discussion and the literature review, we conducted a second structured discussion in October 2015 with 20 UNICEF staff directly involved in the Ebola response at the global, regional, and country level. This discussion incorporated a modified Delphi technique^9^ to gather qualitative responses to open-ended questions about the lessons learned. The discussion group sorted the responses into 21 sub-domains of interventions under 7 main domains of inquiry (Box 1).

BOX 1. **Key Communication Lessons Learned From the Ebola Response in West Africa, Elicited Through a Structured Discussion With UNICEF Staff, October 2015**

**Domain 1. Strategy and Decentralization**

- Decentralization of community engagement
- Funding for community engagement
- Providing supplies for community engagement, such as motorbikes, mobile phones and airtime credits, posters, printing, and radio announcements and shows

**Domain 2. Coordination and Standard Operating Procedures**

- Coordination of community engagement
- Timeliness and relevance of community engagement interventions
- Applying standard operating procedures for community engagement

**Domain 3. Entering and Engaging Communities**

- Entering communities, listening to them, and building their trust
- Community engagement around Community Care Centers
- Interventions for school children and “Back to School” efforts
- Child protection and child-friendly spaces
- Working with survivors, counseling, or addressing stigma/discrimination issues

**Domain 4. Messaging**

- Rumor tracking
- Developing and adjusting key messages

**Domain 5. Partnerships**

- Building broad partnerships for community engagement
- Working with religious leaders
- Working with local journalists and community radio

**Domain 6. Capacity Building**

- Capacity building in community engagement
- Management of staff, including recruitment, training, and ongoing support

**Domain 7. Innovations in Data and Performance Monitoring**

- Data collection and availability
- Research
- Development of indicators for community engagement, or other monitoring and evaluation issues

**Survey:** We then used the 7 domains of inquiry and 21 sub-domains of interventions to frame a voluntary online survey with individuals who worked between July 2014 and April 2015 on Ebola with governments, the UN, or any partner organization in any of the 3 affected countries or in a regional or global support function. Respondents were asked to provide individual opinions and reflections on professional experience, not from an organizational point of view. We asked respondents to rate the perceived success of the 21 interventions using a 10-point scale (1 = “highly unsuccessful”; 10 = “highly successful”)^10^ during 2 phases of the outbreak: (1) when the outbreak was generally increasing in severity (July–December 2014), and (2) when the outbreak was generally coming under control (January–April 2015). We also asked respondents to prioritize interventions in the event of a new Ebola outbreak somewhere in the world and encouraged them to provide additional qualitative information for each question.

In September 2015, we pilot-tested the survey in English and French with UNICEF staff working in the same countries as the target audience and made editorial refinements afterward. We launched the final online survey in October and November 2015 using a snowball technique,^11^ starting with UNICEF and UNMEER staff. No incentives were provided for completing the survey.

**Confirmatory key informant interviews:** Using information from the literature review, the expert discussions, and an analysis of the survey results, we developed a draft set of lessons learned, which we validated through 5 confirmatory key informant interviews with UNICEF and UNMEER senior advisors conducted in December 2015. We also presented and discussed the draft lessons at the International Summit on Social and Behavior Change Communication in Ethiopia in February 2016. In both the interviews and at the summit, partners provided positive and confirmatory feedback on validity of the lessons.

### Limitations

The majority of survey respondents were from UNICEF, with a minority from UNMEER, WHO, NGOs, and civil society organizations, because we used the snowball sampling method. In addition, we collected limited information about the respondents, so it is not possible to determine sex or age differences in the survey responses, nor differences related to position or time spent working on the Ebola response in West Africa. Furthermore, the data collection took place after Ebola was generally considered under control in Sierra Leone and Liberia, although less so in Guinea. Recall bias may therefore be evident given that the data were collected several months after the reporting period of interest. However, we triangulated data from multiple sources to consolidate themes that emerged from more than 1 source, thereby limiting the impact of bias.

## FINDINGS

A total of 53 respondents from UNICEF, UNMEER, NGOs, government, and civil society organizations completed the survey (n = 43 English, n = 10 French). The majority reported working in one of the 3 affected countries during the outbreak: Liberia (n = 23), Guinea (n = 17), or Sierra Leone (n = 9). Five respondents reported working in the regional office and 3 at a headquarters location. (Respondents could report more than 1 duty station.)

**FIGURE 1:**
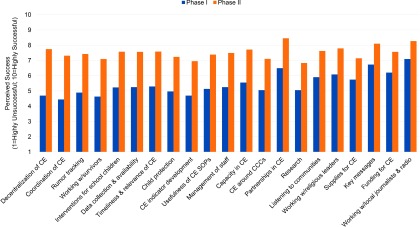
Communication for Development Challenges and Successes in Phase I (July–December 2014) and Phase II (January–April 2015) of the Ebola Epidemic in West Africa Abbreviations: CCC, community care center; CE, community engagement; SOP, standard operating procedure. Source: 2015 survey of UNICEF, UNMEER, NGO, government, and civil society staff who worked on Ebola between July 2014 and April 2015.

According to survey respondents, the 5 most challenging elements during phase 1 of the response consisted of: (1) coordinating community engagement efforts; (2) working with survivors, counseling, or addressing stigma and discrimination issues; (3) developing community engagement indicators or other monitoring and evaluation issues; (4) decentralizing community engagement; and (5) tracking rumors (Figure 1). The elements considered most successful during phase 1 were: (1) working with journalists and community radio; (2) developing key messages; (3) building partnerships for community engagement; (4) funding for community engagement; and (5) working with religious leaders.

Respondents reported that all elements during phase 2 were overall more successful than during phase 1; the 5 most successful elements during phase 2 were: (1) building partnerships for community engagement; (2) working with local journalists and radio; (3) developing key messages; (4) working with religious leaders; and (5) decentralizing community engagement. The most challenging elements during phase 2 were: (1) research; (2) developing community engagement indicators or other monitoring and evaluation issues; (3) working with survivors, counseling, or addressing stigma and discrimination issues; (4) community engagement around community care centers (CCCs); and (5) providing supplies for community engagement. The elements that achieved the greatest improvement between phase 1 and phase 2 were: (1) decentralizing community engagement; (2) coordinating community engagement; (3) tracking rumors; (4) working with survivors, counseling, or addressing stigma and discrimination issues; and (5) implementing interventions for school children.

In future EVD outbreaks, the majority of respondents prioritized coordination (77%), followed by listening to communities and building their trust (45%) and decentralization (30%), as the 3 elements to focus on. This was followed by timeliness and relevance of community engagement (23%), funding for community engagement (21%), and building capacity in community engagement (19%).

After triangulating the findings from all data sources, including the survey findings, expert discussions, and the literature review, we distilled lessons under the following 7 major domains.

1. Strategy and decentralization
2. Coordination and SOPs
3. Entering and engaging communities
4. Messaging
5. Partnerships
6. Capacity building
7. Data and performance monitoring

### Strategy and Decentralization

**Lesson learned:** Establish a comprehensive strategy that focuses on key behaviors, places communities at the center during all phases of the response, and facilitates decentralization with high-quality C4D programming integrated across sectors.

Programmers in the 3 countries affected by the Ebola epidemic used a number of health-related behavior change theories to develop C4D strategies. For example, the socio-ecological model was used from the outset to understand and respond to the individual, community, social, and political dynamics driving EVD.^12^ However, during the first phase of the response, community demand for information and services was not matched with adequate supply, which initially undermined community engagement efforts. These negative community experiences generated mistrust and other barriers to behavior change, which once established were difficult to overcome. The Supply–Enabling Environment–Demand (SEED) model,^13^ which pertains to the influence of supply, the enabling environment, and demand for services on health behavior, was useful in Sierra Leone to bring these factors into balance. Similarly, the Stages of Change Theory was used to address the need for a differentiated response as the outbreak progressed; however, capacity limitations initially inhibited progress.^10^

Identifying influential or trusted sources of information was reinforced as a prerequisite for building community confidence in both rural and urban settings. However, the respondents also suggested that community engagement in rural areas generally required different strategies than in urban centers (Table). For example, in rural communities religious and other community leaders were very influential and had extensive reach, whereas different approaches were needed in densely populated urban settings with diverse information needs and living arrangements, such as informal settlements.

**TABLE:** Community Engagement Considerations in Rural and Urban Settings, Based on Ebola Experience in Guinea, Liberia, and Sierra Leone

Issue/Factor	Rural	Urban
Socio-demographics (e.g., poverty, literacy, education)	Approaches need to be tailored to socioeconomic status and literacy, but can be managed.	Literacy tends to be higher and English understood more than in rural settings, but still difficult to cater for the diversity in socioeconomic status in densely populated urban settings.
Traditional, social government structures that provide potential for sustainability, but can sometimes marginalize groups of people or other times provide an opportunity for better reach	High	Low
Understanding and correcting rumors	Localized rumors can be settled with local leaders and/or in a community meeting more easily than in urban areas, but still hard if various rumors are circulating.	Very hard to correct misinformation once widely circulated. Mistrust tends to fuel further distortion and undermine efforts to correct misinformation.
Access and reach for supplies and logistics	Easier to distribute than in urban areas, although further away.	Hard to distribute due to congestion/population density.
Partner coordination between regional and local command centers	Very organized and responsive, once up and running.	Hard to cope with very high demand; needs additional contingency and resources.
Data collection and monitoring	Hard because communities can be cautious and it is hard to reach everyone.	Hard due to dense population, difficult living conditions, lack of trust. Data collection and feedback are usually too slow to keep pace with changing situations in communities.
Differences in Preparation, Response, and Recovery phases	Initially Ebola was concentrated in rural areas; response improved with decentralized command centers.	As Ebola intensified, it also reached urban areas and the response struggled to keep pace. Many areas had no prevalence for a long time. Hard to remain vigilant over protracted period.
Interpersonal vs. mass media communication approaches	Mass media (radio) worked well in rural areas (when tailored regarding language, messenger, etc.), with reinforcement from interpersonal approaches (e.g., chiefs, religious leaders, community groups).	Mass media in urban areas is hard to tailor to all needs; interpersonal approaches are very labor intensive for urban settings.
Incentives	Hard; incentives need to be set out clearly across organizations and functions, and consistently followed everywhere, from chiefs to volunteers.	Hard; consistency across organizations and administration is very complicated in densely populated areas.
Capacity of health staff, community mobilizers, and ability to work together in teams	Hard to recruit and support the full range of technical and management skills, local and international staff, etc., especially for long periods.	While more people are available in urban settings, it is still hard to recruit and support the full range of skills needed, especially for long periods.

The need to adapt quickly to different contexts requires a decentralized approach to C4D programming. Respondents highlighted this importance and stressed that strategies need to address the complexities of community and cultural hierarchies and other local factors from the outset. As the Ebola response as a whole matured, including the C4D components, success was achieved through greater focus on customary burials and predicting related hot spots. At the same time, it was recognized that the approach must be tailored to the context. For example, different information and actions were required for different groups, such as survivors, pregnant women, or fishing communities,^14^ and these efforts needed to be well coordinated with community expectations regarding, for example, supplies in quarantine situations or safety concerns regarding “Back to School” initiatives during the later phase. It was not until several months into the epidemic that the national response in the 3 countries had the capacity, coordination mechanisms, and sub-structures in place to manage the necessary decentralized approach.

*Decentralization/community ownership including availability of funding and proper coordination of intervention activities were critical catalysts that facilitated the successful eradication of the Ebola Virus in Liberia.* (Survey respondent)

### Coordination and Standard Operating Procedures

**Lessons learned:** Establish solid C4D leadership at all levels with the necessary authority to coordinate partners. Introduce and enforce SOPs for C4D from the outset as a central coordination and quality assurance tool. Dedicate attention to coordination capacity to manage decentralization of the C4D response and resources across all sectors, as well as to detailed planning during all stages of the response.^15^

Respondents suggested that a lack of high-level, trusted leadership during the early phase of the response delayed effective roll out and coordination of C4D across the response and identified effective leadership and coordination of the many partners working on C4D as central to overcoming challenges more quickly and improving the quality of programming. As the response decentralized to districts over the course of the epidemic, the need for clear leadership and strong protocols to guide all aspects of the response strategy with consistency was underscored even more, from how to enter communities, to micro-mapping of communities, to accurate data collection and beyond. Although local decision making can be more flexible and responsive to the local context, in the absence of SOPs, implementation can become fragmented and ineffectual. Therefore, a delicate balance needs to be achieved between an approach that is flexible, responsive, and decentralized on the one hand and well-coordinated, consistent, and streamlined on the other hand. Respondents noted that SOPs provided the necessary authority to demand that C4D be integrated and paired with other sectors and pillars to unify the emergency response,^16^ and they reiterated the need for SOPs on C4D to be available early—to all partners—and enforced to improve the quality and consistency of C4D.

*I learned the challenges of coordination: each partner want[ed] to rule. Partners are not working hand in hand and are more likely to promote their own agendas than fighting the outbreak to release overwhelmed communities. It is important to recognize the capacity, strengths and competencies of others … good coordination has shown its relevance in producing harmonized messages, joined and strong SocMob [social mobilization] campaigns, etc. Decentralizing the community engagement must be planned from the beginning of the outbreak to ensure [the] local level is truly involved and national level strategies are taken to [the] community level.* (Survey respondent)

*… without coordination of partners … there will be duplication of others' work …* (Survey respondent)

**FIGURE 2:**
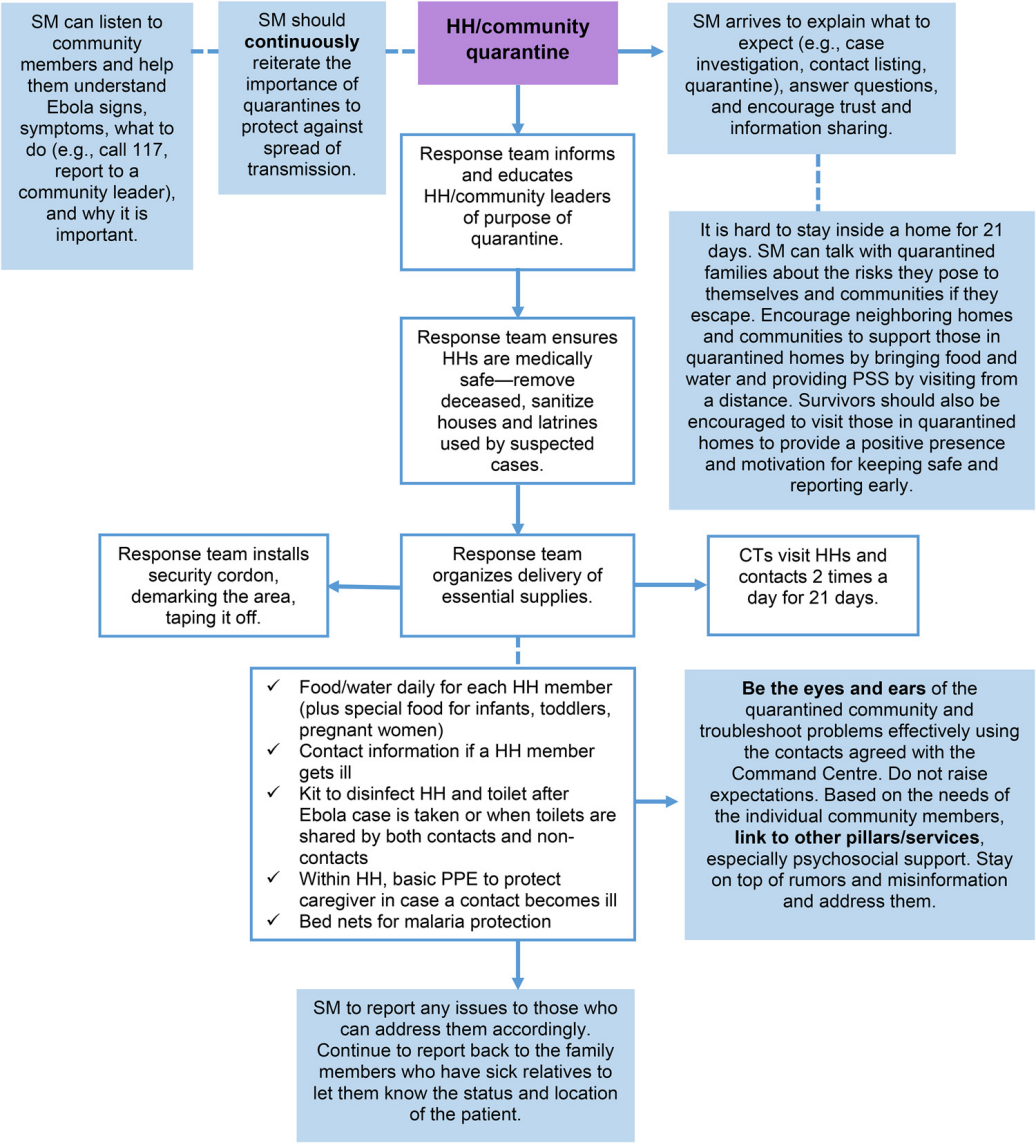
Integration of Social Mobilization Into Quarantine Protocols Abbreviations: CT, contact tracer; HH, household; PPE, personal protective equipment; PSS, psychosocial support; SM, social mobilization. Source: National Ebola Response Centre 2015.^17^

Ideally, all implementing partners would endorse the SOPs and conduct standardized training as a requirement for their personnel to participate in the formal response. While dedicated technical expertise in C4D is absolutely central, all sectors would benefit from improving skills in engaging with communities to create harmonized ways of working. For example, social mobilizers can be paired with surveillance officers and active case finders or quarantine teams (Figure 2) or female social mobilizers can be included in ambulance teams, especially when they are attending to female patients. SOPs will need to be routinely reviewed and adapted according to epidemiologic findings and other contextual factors.

**Figure:**
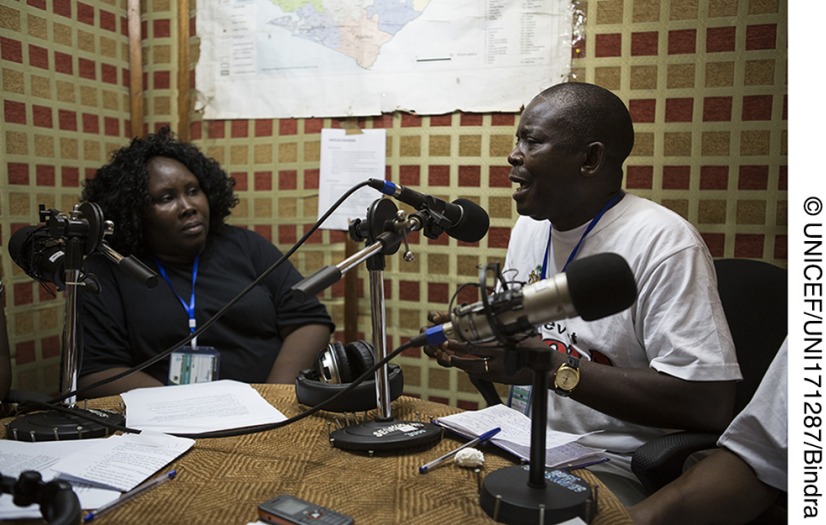
In September 2014, Edmond Bankiu (right), an HIV/AIDS specialist with UNICEF also serving as a focal point for social mobilization efforts during the Ebola outbreak in Sierra Leone, broadcast information about the Ebola campaign via radio in Freetown with one of the hosts (left) of the radio segment. In the 3 countries affected most by Ebola (Guinea, Liberia, and Sierra Leone), radio had the greatest reach and flexibility of all available communication channels.

**Figure:**
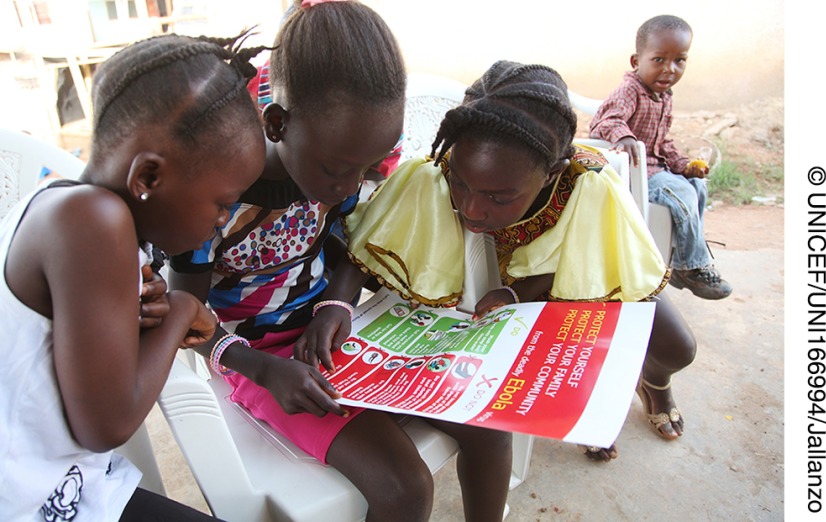
Girls from Lofa County, Liberia, read a poster on how to prevent spreading Ebola. Dissemination of key messages was recognized as one of the stronger elements of the Ebola response in West Africa.

**Figure:**
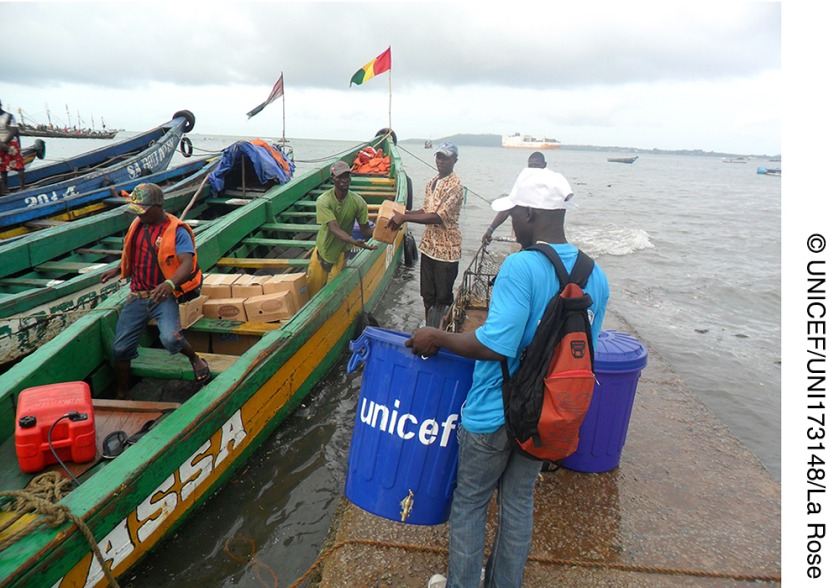
UNICEF staff prepare to travel to islands off the coast of Conakry, Guinea, in May 2014 to share key messages with communities on the symptoms of Ebola and how to prevent its transmission. A global network of communication specialists is needed to support future public health emergency situations.

### Entering and Engaging Communities

**Lessons learned:** Invest in trusted local community members as mobilizers and strengthen broader community systems for long-term resilience.^18^ Identify key influencers and channels of communication with strong reach and relevance while considering more specialized communication for specific sub-groups.

Developing and using the knowledge, attitudes, and C4D skills in communities themselves to shape local solutions is critical, and it requires investment over time and genuine partnership, as well as openness to listen to communities and to take appropriate action in a timely manner. In fact, many communities and local organizations were already taking action to prevent and manage Ebola as the formal response was being developed.^5^ Building on existing community engagement platforms, such as the strong networks of religious leaders in West Africa, was integral to gaining entry and trust in communities. In some communities in Liberia, community engagement for the purpose of addressing open defecation was already in place well before Ebola hit. These communities were able to quickly transfer those skills to Ebola, suggesting that a small amount of additional support can significantly leverage existing community investments.

*From my experience in Liberia, I see community engagement as key to fighting any other outbreak … our people believe in their religious/spiritual/traditional leaders so much that they believe anything they [the leaders] say to them. Once funding is available [and] the communities are engaged to have knowledge and take on the fight themselves, getting any outbreak under control is possible … for instance, Grand Cape Mount, a highly Islamic county, was able to defeat Ebola only after UNICEF engaged religious, traditional, and community leaders to take the key Ebola messages to their own people, thereby creating trust and understanding, which promoted acceptance of early treatment and care-seeking behavior.* (Survey respondent)

In some cases, lack of coordination led to unannounced entry of outsiders to communities, which created anxiety. As noted in a reports by the Overseas Development Institute^7^ and Institute of Development Studies^19^:

The Ebola outbreak could be described as an epidemic of mistrust: the flame of a virus hitting the tinder of suspicion.

Popular suspicion of the motives of foreign organizations and government is rooted in a long history of slavery, civil war, extraction, and, more recently, commercial and noncommercial foreign development efforts [that are] often diverted into the pockets of government and non-governmental organization (NGO) officials.

Local mobilizers—that is, existing community members who have been trained and supported in C4D—will have clearer insight and more community trust and sustainable networks than outsiders. They are essential for achieving genuine community ownership and influencing key behaviors such as care seeking, infection control, and burial practices. Local knowledge is also critical to effective surveillance, contact tracing, and other key aspects of the response. An UNMEER consultation report emphasized 3 particular areas where local mobilizers played a key role: (1) overcoming community resistance, (2) understanding local context, such as sociocultural norms, decision-making processes, urban and rural considerations, and cross-border issues, and (3) transition and emerging issues such as getting back to school, including vaccine trials and routine immunization.^20^

*Why is the debate being reopened on engagement? It is not new and has worked in many contexts over years … [but still] we failed to solve the local conflict because the solutions were not coming from the community itself.* (Survey respondent)

*Community engagement is an art and a science—forget precision. Community engagement takes time and we have to invest time, resources to gain respect/influence of the community.* (Key informant, civil society)

In terms of channels of communication, in all 3 countries radio was acknowledged as having the most effective reach and greatest flexibility regarding languages and messages. Radio also facilitated 2-way communication with local leaders and networks through call-in sessions, which included religious leaders, chiefs, healers, mayors and councilors, and other community leaders. As effective as these channels were, men usually held these positions of authority. The experiences of Ebola survivors, including children, women, those with disabilities, and marginalized groups are distinct and require specific attention. For example, many children were left without caretakers when adults in the household fell ill. Pregnant women were stigmatized because of the potential for infection during delivery. Therefore, a mix of communication channels with tailored messages is essential.

### Messaging

**Lesson learned:** As the patterns of the epidemic change over time, continually adapt messages and strategies that are most relevant to communities' understanding of the health issue, to their information needs, and to what is most likely to prevent and control infections.

The dissemination of key messages was highlighted by respondents as one of the stronger elements of the response, especially in terms of basic knowledge of Ebola prevention practices. Four key desired behavioral outcomes were consistent across all 3 countries throughout the Ebola response: (1) prevention practices including hygiene and handwashing, (2) case and contact reporting, (3) safe and dignified burials, and (4) early treatment and care seeking (Box 2). However, qualitative comments from the structured expert discussions and survey suggested that greater attention to the evolution of messages over time was needed. During the early stages of the epidemic, messages that focused on Ebola causing death and no cure being available frightened communities. Similarly, information toward the end of the epidemic that the virus could remain in body fluids for several months after recovery fueled stigma against survivors. These negative messages were perceived as driving people away from organized services and toward untested remedies.

BOX 2. **Key Desired Behavioral Outcomes in Response to the 2013–2016 Ebola Epidemic**

Desired behavioral outcomes for prevention, detection, and treatment of Ebola Virus Disease were consistent across Guinea, Liberia, and Sierra Leone—the 3 countries most affected by the 2013–2016 Ebola epidemic. The behavioral outcomes spanned the following 4 main categories:

- Hygiene, handwashing, and other infection control practices
- Safe and dignified burials
- Case and contact reporting
- Early treatment and care seeking

See the Appendix at the end of this article for sample communication materials used in each country to address these desired outcomes. Additional samples are also provided as supplementary materials.

While simplicity in messaging is important to understanding, oversimplified messages that did not provide sufficient information were widespread, especially as the epidemic progressed. Unhelpful rumors circulated in all 3 countries and confounded efforts to convey facts or clarify what was needed for and from communities. Survey respondents spoke of examples of people with suspected Ebola being transported in ambulances without adequate feedback to the community about where the patients were taken or how to receive updates on their condition. This lack of communication supported rumors that ambulances were a source of infection. Rumors spread quickly, also highlighting the need to match community demand for information with high-quality, well-communicated services to capture and maintain trust. Furthermore, communities complained of the lack of services for non-Ebola matters, such as antenatal care, malaria treatment, and services for heart conditions or other ailments. As the response matured, however, the approach became more proactive, particularly by using information from communities to more directly shape messaging and other interventions.

Recognizing these challenges, governments in all 3 countries coordinated with the U.S. Centers for Disease Control and Prevention (CDC), UNICEF, and other partners to improve information flow and address rumors through weekly updates around evolving themes. These messages were then synchronized with radio communication, religious sermons, and other community channels. For example, as more survivors returned to the community, more emphasis was placed on the reintegration and support of survivors. In communities where this mobilization and support was consistent, this facilitated survivors resettling into communities.

*Listening to communities and building trust is the key to the success of community engagement strategies. We need to know the community and need them to trust us … [also] socio-anthropological expertise is very relevant.* (Survey respondent)

### Partnerships

**Lesson learned:** Invest in strategic partnerships to achieve short- and long-term goals, starting with communities themselves, to build strategies, skills, and other resources that are most relevant to community understanding of the health issue and to controlling the outbreak.

Survey respondents rated partnerships with community, religious leaders, journalists, and radio stations as key elements of success throughout the response, reinforcing the principle of communities being the central partner in C4D. Coordination among partners was also noted as critical, particularly given the large number of international and local NGOs involved in C4D and the nature of donor and government relationships. For example, in Liberia 76 partner organizations organized 830 public health trainers who trained 15,000 community educators. These community educators equipped more than 2 million Liberians with lifesaving information about how to protect themselves and their families from Ebola.^21^ It is important to note that a number of organizations withdrew from the affected countries as the Ebola outbreak spread while new organizations emerged on the scene and many new staff arrived. This created enormous challenges in coordination and establishing trust with different partners.

In reflecting on the first phase of the outbreak, survey respondents mentioned overemphasis by partners in all 3 countries on producing simple materials (e.g., posters, flyers) to convey key messages, such that some of these resources may have been better applied to more complex tasks including more intensive community engagement.

*Some partners have put a lot of money in developing materials (posters, flyers, banners). However, no one among these partners has courage to assess the impact of these materials. Or even determine what could have been the right quantity to reproduce. Thousand[s] of [materials] are stored at the airport.* (Survey respondent)

Respondents indicated that coordination among partners significantly improved during the second phase, and partnership mechanisms, such as working groups, that were established to manage key messages, coordination, research, and other tasks became more efficient. Micro-mapping of communities was also conducted to improve targeting, with agreed division of labor from partners across geographical areas. This activity both required and built high levels of coordination and trust in these partnerships. These mechanisms and the pressure to use existing capacity wisely also imposed a level of discipline to engage only those partners who were necessary to the particular task.

### Capacity Building

**Lesson learned:** Establish and support a network of local and international professionals with capacity in C4D, including both management and technical skills, who can be deployed rapidly and remain in place for significant amounts of time to supplement national systems.

Challenges in attracting and maintaining personnel with adequate capacity over time is commonly reported in emergency situations, as was the case during the Ebola outbreak. Many international organizations deployed staff for only weeks at a time, especially during the early phase. The high turnover of staff undermined continuity and frustrated coordination efforts. In addition, in the case of C4D, there was an insufficient range of capacities, resulting in too much “megaphone-style” mass communication and too little comprehensive health promotion and behavioral science, coordination, leadership, management, and strategy capacity. Finally, the sheer volume of organizations engaged in C4D to varying degrees in the 3 countries—more than 30 international and many more local organizations—required very different skills related to central management compared with the technical skills required for fieldwork in urban, rural, or remote areas.

In the future, staff should be provided with a common orientation, trained in agreed SOPs and minimum standards (including safety), and given ongoing support, including supportive supervision. In addition to improving the quality and consistency of the emergency response, these measures help to keep staff healthy and to reduce turnover, especially considering the stress that emergencies can impose.

As part of efforts to formalize C4D as an integral element of the global humanitarian infrastructure, a global network of C4D specialists is needed to support the surge capacity, along with standardized procedures to address administrative issues, predeployment training, and fast-track recruitment. Harmonization of incentives and compensation to mobilizers at all levels, across organizations, and regardless of whether they are international or local should also be undertaken.^7^^,^^22^ Dr. Tom Frieden, Director of the CDC, commented on the need for capacity development to rapidly detect and respond to future outbreaks^18^:

We need rapid-response teams; one of the things we did in Liberia was to implement rapid-response capacity, so that when cases emerged in rural areas we sent a team out immediately and they were able to stop the virus within one or two generations of it. We need increased prevention wherever possible.

### Innovations in Data and Performance Monitoring

**Lesson learned:** Establish clear C4D process and impact indicators and an accessible harmonized data platform for monitoring, and strive for innovations in real-time data analysis and rapid feedback to communities and authorities to inform decision making.

There was a range of qualitative and quantitative data sources in the formal response, including field-based observational data, adapted mobile phone platforms, call-center data, and nationally representative surveys. In addition, individual partners conducted small- and large-scale studies, evaluations, and other reviews. Despite providing valuable information, synthesis from these various sources and dissemination of the data were inadequate and could not keep pace with the outbreak. Furthermore, because C4D is process-oriented, it was initially difficult to agree on useful indicators that could be applied across the affected countries.

Despite challenges, impressive achievements also emerged. Sierra Leone completed 3 nationally representative surveys of knowledge, attitudes, and practices (KAP) over 7 months of the outbreak, which provided strong evidence that C4D was having an impact, and was critical to improving decision making and program strategy.^23^ Innovations in open-source platforms for mobile phones, such as Rapid Pro and U-report,^24^ as well as mobile messaging (SMS) were deployed across all 3 affected countries to gather real-time community insights and attract underrepresented groups such as young people. These technologies enabled greater responsiveness to rumors that required rapid redress to prevent undermining the response.^4^

Increasing partner access to common monitoring platforms with real-time analysis and clear feedback mechanisms to communities is *essential* to managing future outbreaks. There is also a need to agree on predefined C4D indicators and mechanisms, including strong coordination of key activities and monitoring of the response among partners. Establishing a monitoring and evaluation plan from the outset of the emergency to support the overall C4D strategy is also essential. Such a plan must be informed by a range of data, including anthropological, epidemiological, qualitative, and quantitative data, and show greater respect for community perceptions and rumors.

*Difficult to reach scale at reasonable cost … Difficult to integrate [indicators] across sectors, partly because each sector wants to include so much detail. Also difficult to measure impact and cost-effectiveness [of C4D].* (Expert discussion participant)

*Rumor tracking is also key; many people lost their lives because of rumors, myth, denial, etc.* (Survey respondent)

## OUTLOOK FOR FUTURE OUTBREAKS (AND OTHER EMERGENCIES)

Despite early warning signs, the Ebola outbreak took the world by surprise. The lack of preparedness, lack of acknowledgment of the potential spread of Ebola, and delays in funding resulted in a race to catch up to the virus, rather than getting ahead of it from the start. Each of the 3 most affected countries struggled to simultaneously implement a myriad of approaches to address the varied challenges emerging in different parts of the country. As noted in a report on the Ebola response by the Overseas Development Institute^7^:

Ebola exposed much about the international aid community: [it was] dedicated, resourceful, and diverse, as well as ill-prepared, donor-dependent, and tested by the confrontation between technical approaches and the complexities of the sociocultural context.

The lessons learned from the Ebola response in West Africa, particularly the C4D response, are based on a very specific context: the situation was rapidly unfolding and full of surprises and the communities that were affected the most were largely low-income and remote, and they often held traditional practices and rituals that were difficult to change. Nevertheless, the basic principles uncovered from the Ebola response can be applied to future disease outbreaks, not least the need to focus on prevention as well as treatment. Furthermore, some of the lessons that emerged from this analysis, including engaging communities early on, understanding social and behavioral dynamics to shape the response, adapting to the evolution of the epidemic and to feedback from communities, and facilitating a more central and active role of communities with mutual accountability mechanisms, have been well known for some time and should not have been overlooked. Specifically, various conceptual and theoretical models have been applied in health and development programming, including in emergency responses, to better address social and behavioral dynamics. For instance, the socio-ecological model posits the need to understand drivers of behaviors and change across different domains of influence—from individual and interpersonal to community and social and political—which may require different types of communication and engagement.^12^ Similarly, the Stages of Change Theory has long espoused the need for different information and approaches as people and communities move through different stages in their experience of a health-related issue.^10^ At the same time, many of the “right things” were included in national strategies responding to the Ebola outbreak; implementation and adaptation over time, however, proved difficult.^25^ It was not until the response acknowledged the essential need for effective coordination and SOPs, integration of C4D with other components of the response, and enhancing local capacities that progress started to happen.

While differences exist, many similarities in lessons learned have been drawn from the Ebola outbreak in West Africa and other events, such as earthquakes in Haiti and Nepal, the Zika outbreak in Latin America, and longer-term challenges such as HIV or polio. The undeniable social dimensions of these public health issues highlight the centrality of community engagement as well as the wider implications of social and behavior change. We can look to the recent Zika outbreak as a specific case example of how lessons from the Ebola response also apply to this situation even though the virus, including transmission, symptoms, and treatment, are considerably different from Ebola. In early 2016, WHO declared a Public Health Emergency of International Concern due to the strong association between Zika virus infection during pregnancy and an increase in cases of microcephaly as well as other congenital complications, particularly in Brazil and other countries in Latin America. Because Zika is transmitted by the same mosquito that transmits dengue—an endemic disease in many Latin American countries—prevention efforts focused on engaging local communities to minimize exposure to the vector and to promote uptake of preventive behaviors including use of bed nets. As with Ebola, the Zika response has evolved from a primary focus on prevention to additional efforts to provide care and support to affected families. These developments require that communication and community engagement activities be flexible, adaptable, well-coordinated, and guided by data and evidence.

Communication, community engagement, and social mobilization proved their value to the individual and community behavior change objectives of the Ebola response. However, they are somewhat new to the global health emergency context and thus there is a need to formally place these approaches within the global humanitarian response architecture. Implementing organizations need to strengthen their capacity to fulfill C4D accountabilities as part of the formal cluster system, including clear SOPs, tools, training, and ongoing support. The Ebola experience also shined a strong light on the need to strengthen governance and accountability and wider systems strengthening, such as data systems, standardized indicators, supply chain, and use of real-time technology, ideally through a common platform. Strengthening capacities of national and local governments to effectively address these types of emergencies, including a focus on C4D and risk communication, should be an important component of these efforts, which in turn could lead to greater accountability. This has been highlighted in multiple assessments, reports, and studies including the WHO Assessment of the Ebola response.^26^ These enhancements are already underway in the affected countries and elsewhere, in terms of risk-informed public health and resilience programming, including preparedness and readiness for potential public health threats—but they certainly will require long-term investments and focus. Ultimately, future success relies on a fully functional C4D coordination mechanism within the formal humanitarian infrastructure that is supported across the board and funded accordingly. This will be key to fulfilling the vision of the new “Grand Bargain” for humanitarian action endorsed at the 2016 World Humanitarian Summit,^27^ which seeks to more decisively put people and affected communities at the center of any response.

All of the above will require more predictable funding. Learning from other long-standing cross-cutting issues has shown that firm management agreements and practices are required. One possible approach, common in some fields such as evaluation and gender equity, involves designating a specific percentage of sectoral funds, likely between 10% and 20%, to support C4D efforts. Without formal action on such policies, the important benefits derived from social mobilization will remain ad hoc.

UNICEF has taken important steps to respond to lessons outlined in this article. Two important initiatives with implications for the broader humanitarian sector are worth highlighting. First, in coordination with other UN agencies, the Communicating with Disaster Affected Communities (CDAC) Network and other key stakeholders, UNICEF plans to establish a communication and community engagement platform within the global humanitarian architecture that will provide rapid access to surge capacity, greater predictability of response, common standards and tools, and clearly defined roles and responsibilities among humanitarian actors. The initiative will require long-term investments and funding linked to preparedness efforts. Second, UNICEF also plans to establish a global platform that will facilitate rapid synthesis of existing evidence and anthropological data that can quickly inform community engagement strategy and action. This platform will function as a global help desk, which will (1) identify and synthesize in advance relevant data and evidence on social and behavioral dynamics related to emergency response (e.g., engaging pastoralist communities in public health emergencies), and (2) respond to specific requests for available data and evidence in ongoing emergency situations. These efforts are not limited to public health emergencies but will function across different types of emergencies and humanitarian situations.

## CONCLUSION

“Political and financial dynamics create a tendency towards cure, rather than prevention,”^7^ stated an Ebola evaluation report. However, in Guinea, Liberia, and Sierra Leone, a realization came about that Ebola is as much a social issue as a health issue, and, along with that, the countries realized the value of early, genuine engagement with communities.^28^ This is in essence the crucial lesson learned from the Ebola outbreak—one that should be carried forward, for when the legitimacy of C4D is recognized across sectors from the outset and organizations expand community systems fully, a range of issues beyond the specific emergency at hand will be supported effectively.

## Supplementary Material

supplementary materials

## Notes

**Peer Reviewed**

## APPENDIX.

Sample Communication Materials Used in the 2013?2016 Ebola Response

The following samples are provided in English but were available in many languages. These samples are shown for illustrative purposes only and should not be considered better or worse than other materials that were used in the countries affected by Ebola.

See the supplementary materials for additional samples.

**Hygiene, Handwashing, and Other Infection Control Practices (poster from ActionAid, Liberia)**

**Safe and Dignified Burials (poster from the Ministry of Health, Sierra Leone, and the U.S. Centers for Disease Control and Prevention)**

**Case and Contact Reporting (poster from the U.S. Centers for Disease Control and Prevention, Sierra Leone)**

**Early Treatment and Care Seeking (poster from the U.S. Centers for Disease Control and Prevention)**
