# Supplementary material for: Social Mobilization and Community Engagement Central to the Ebola Response in West Africa: Lessons for Future Public Health Emergencies
Source: Glob Health Sci Pract. 2016 Dec 23;4(4):626–46. doi: 10.9745/GHSP-D-16-00226 (PMC5199179; doi:10.9745/GHSP-D-16-00226)
Supplement: supplementary materials [file GHSP-D-16-00226_index.html]

Supplement to Social Mobilization and Community Engagement Central to the Ebola Response in West Africa: Lessons for Future Public Health Emergencies | Global Health: Science and Practice

## Supplementary Data

Supplementary Data

- Text s01, JPG - Text s01, JPG
- Text so2, PDF - Text so2, PDF
- Text so3, PDF - Text so3, PDF
- Text so4, PDF - Text so4, PDF
- Text so5, PDF - Text so5, PDF
