# Supplementary figures and images for: Social Mobilization and Community Engagement Central to the Ebola Response in West Africa: Lessons for Future Public Health Emergencies
Source: Glob Health Sci Pract. 2016 Dec 23;4(4):626–46. doi: 10.9745/GHSP-D-16-00226 (PMC5199179; doi:10.9745/GHSP-D-16-00226)

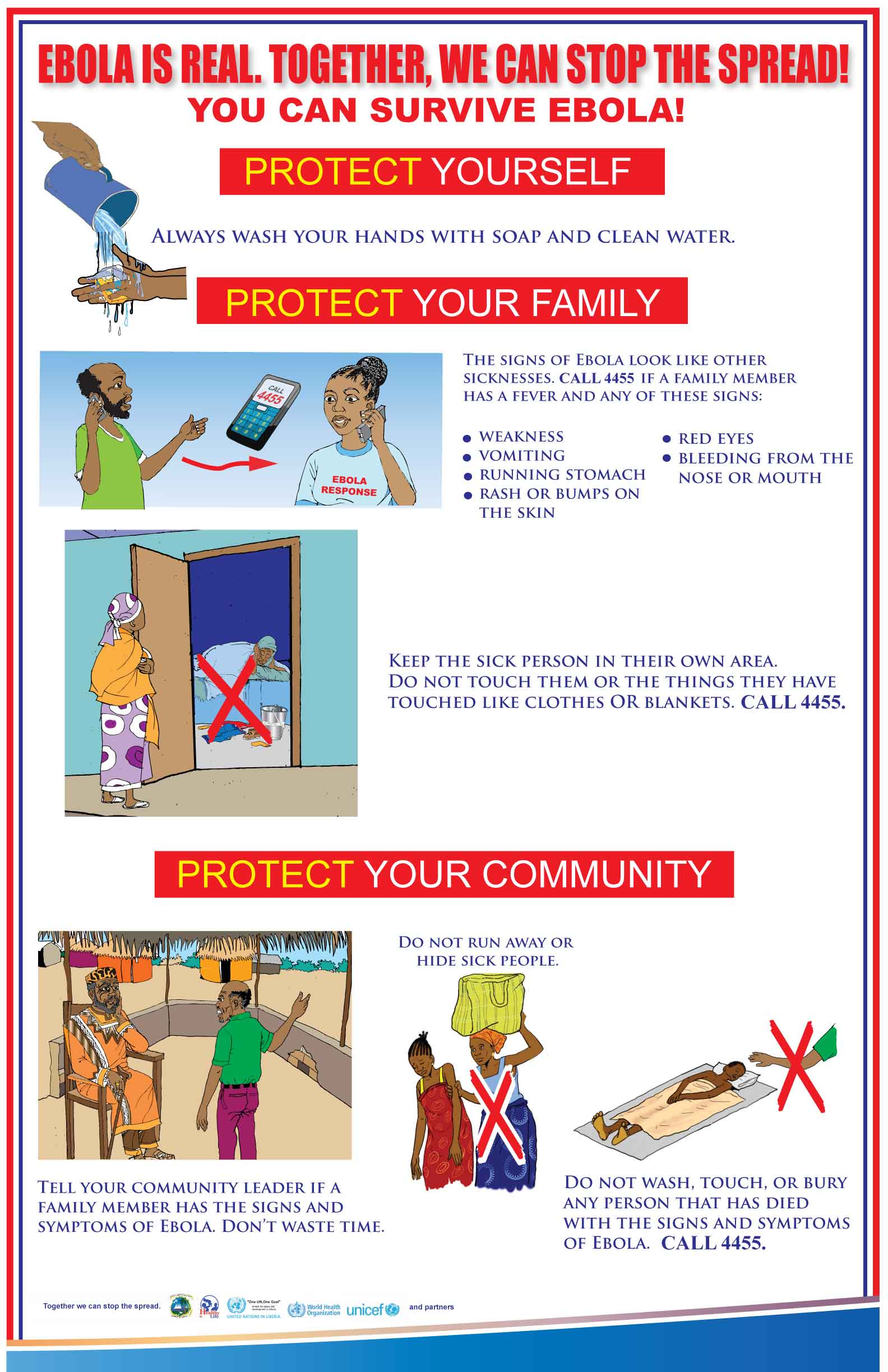

Supplement: supplementary materials [file Supplementary_material-1.jpg]
