# Supplementary material for: Social Mobilization and Community Engagement Central to the Ebola Response in West Africa: Lessons for Future Public Health Emergencies
Source: Glob Health Sci Pract. 2016 Dec 23;4(4):626–46. doi: 10.9745/GHSP-D-16-00226 (PMC5199179; doi:10.9745/GHSP-D-16-00226)
Supplement: supplementary materials [file Supplementary_material-2.pdf]

PROTECT YOURSELF
PROTECT YOUR FAMILY
PROTECT YOUR COMMUNITY

Against the Ebola Virus Disease

DO	DO NOT
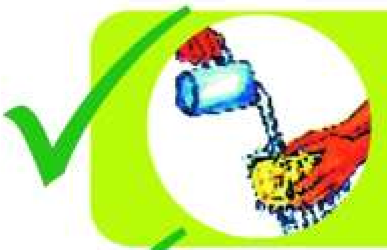 Always Wash your hands with soap and clean water	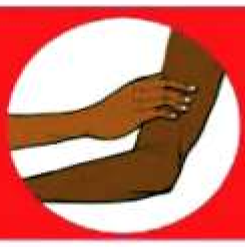 Do not touch people with signs of or have died of Ebola
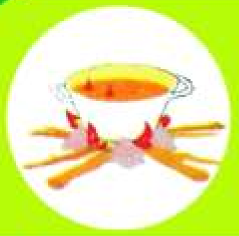 Always cook your food properly	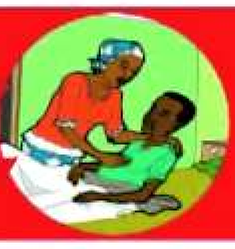 Do not touch clothes & bed cloths of people Who have died of Ebola
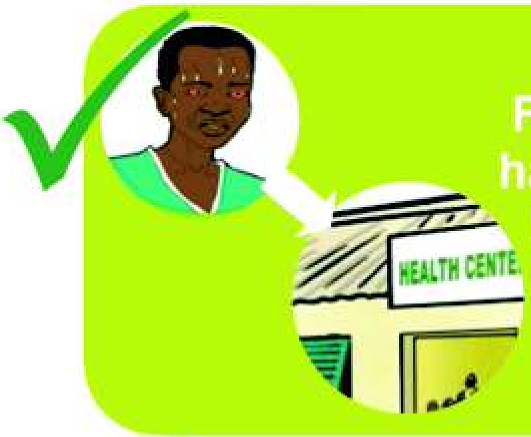 Go to the Health Facility anytime you have headache, Pain, Diarrhea, red eyes rash and vomiting	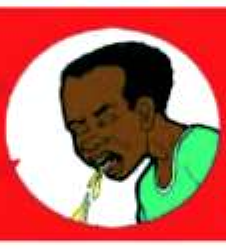 Do not touch vomit, Saliva, Urine, blood and poo poo of people who have signs and symptoms of Ebola
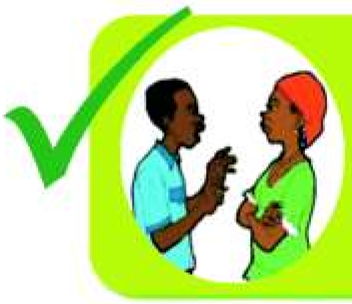 Tell anyone you meet about Ebola so they can be informed	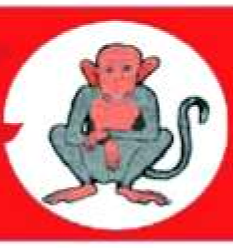 Do not play with Monkeys and Baboons
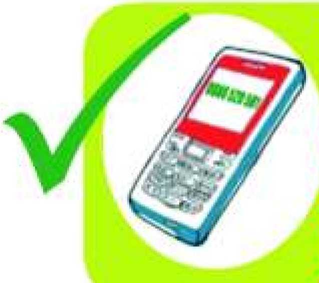 Call these numbers if you need help for help : 0886229641 0886397381 0776547437 1333 (Lonestar) 4455 (Cellcom)	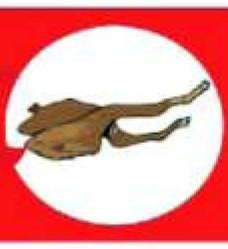 Do not eat bush meat
	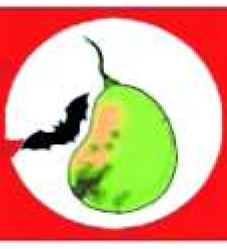 Do not eat plums eaten by bats

Let's stop the spread of Ebola Together

Healthy Life

actionaid Liberia

phil

Empowering to Save Lives.
