# Supplementary material for: Social Mobilization and Community Engagement Central to the Ebola Response in West Africa: Lessons for Future Public Health Emergencies
Source: Glob Health Sci Pract. 2016 Dec 23;4(4):626–46. doi: 10.9745/GHSP-D-16-00226 (PMC5199179; doi:10.9745/GHSP-D-16-00226)
Supplement: supplementary materials [file Supplementary_material-3.pdf]

Ebola Must Go: Bury All Dead Bodies Safely-Call 4455

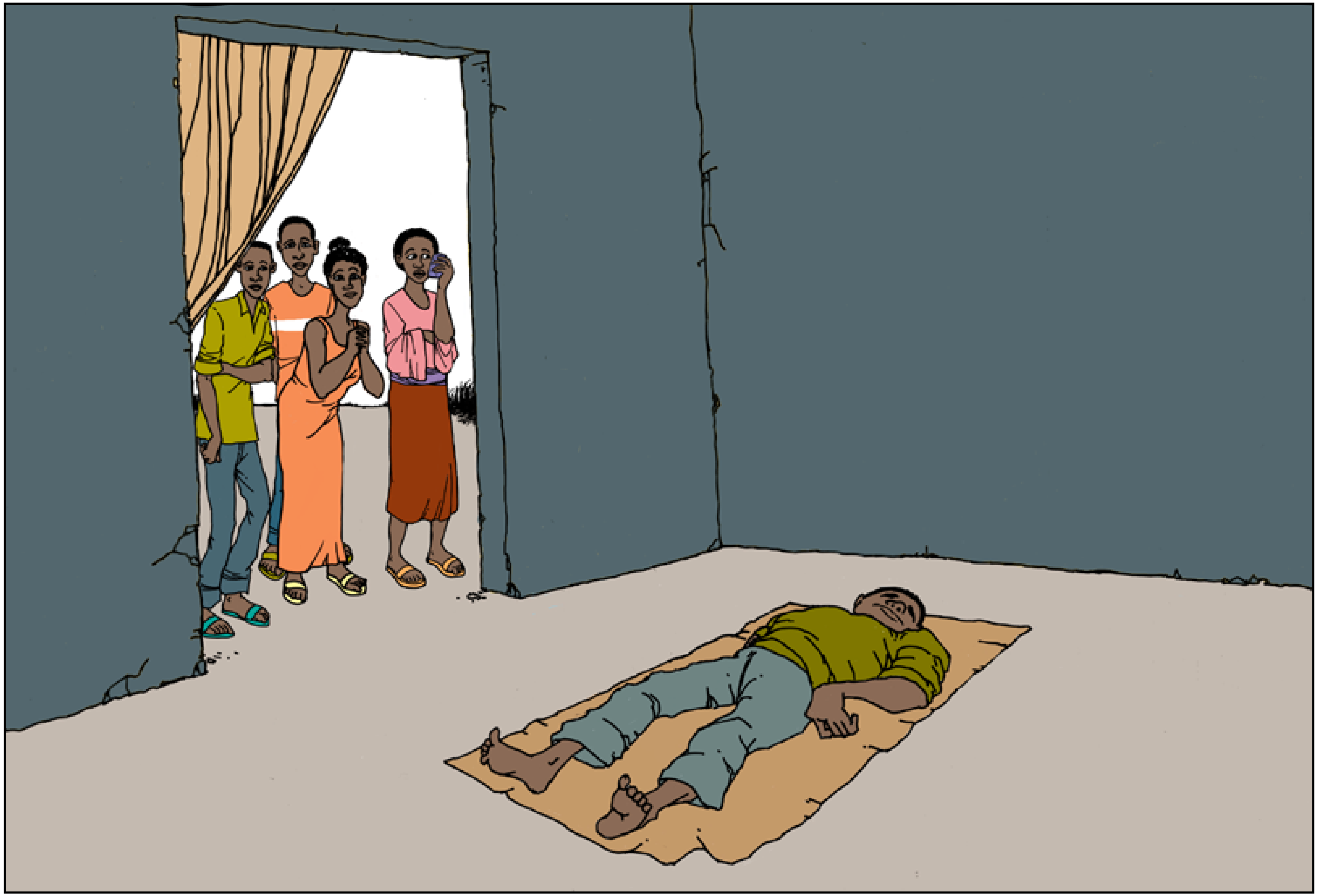

Ebola Must Go: Bury All Dead Bodies Safely: **Call 4455**

12/23/2014 CS252674-C

Ebola Must Go: Bury All Dead Bodies Safely-Call 4455

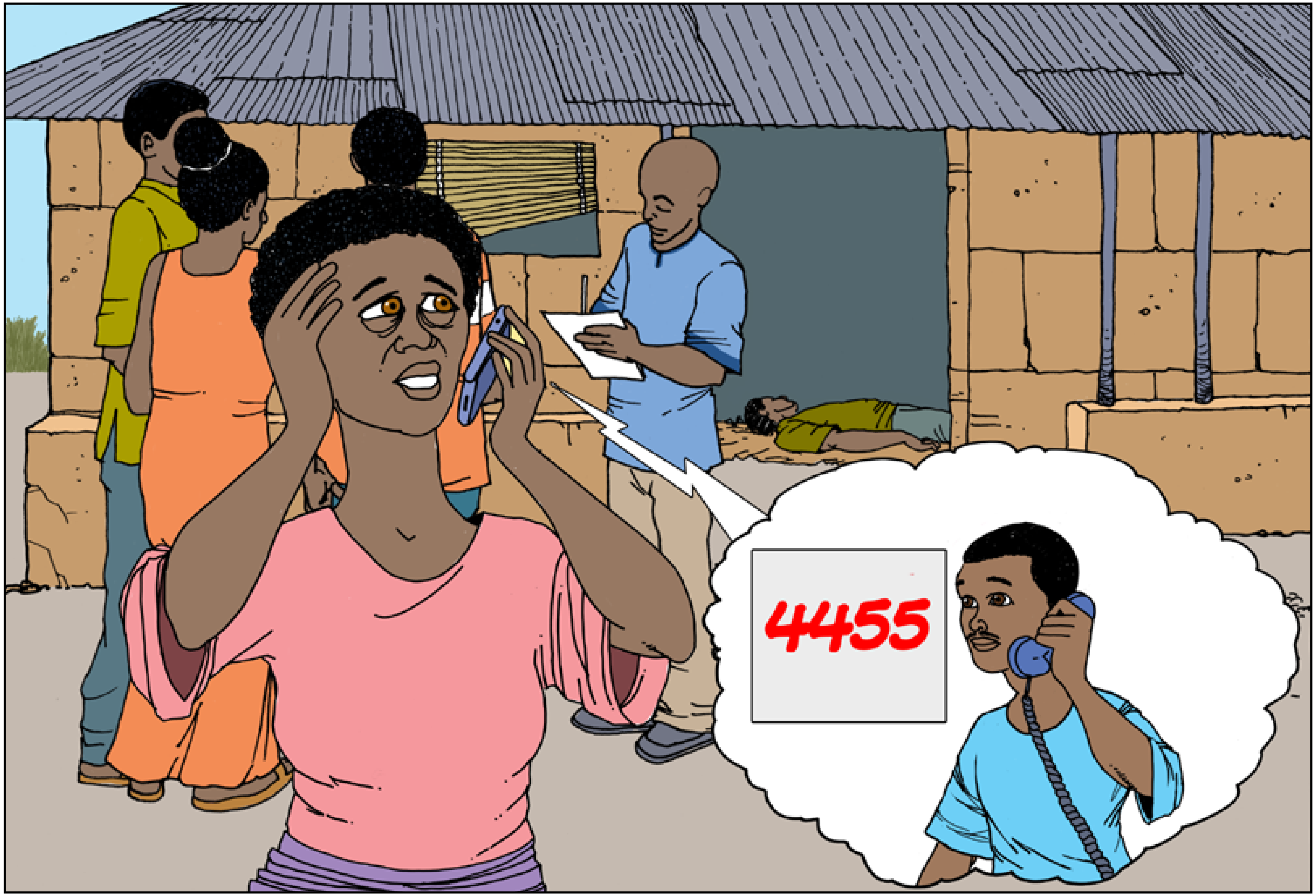

During this Ebola time, we cannot care for family and friends that die the way we are used to. While you are waiting for the burial team to arrive, keep a distance of at least 3 feet (1 meter) from the body. Do not touch it.

Ebola Must Go: Bury All Dead Bodies Safely-**Call 4455**

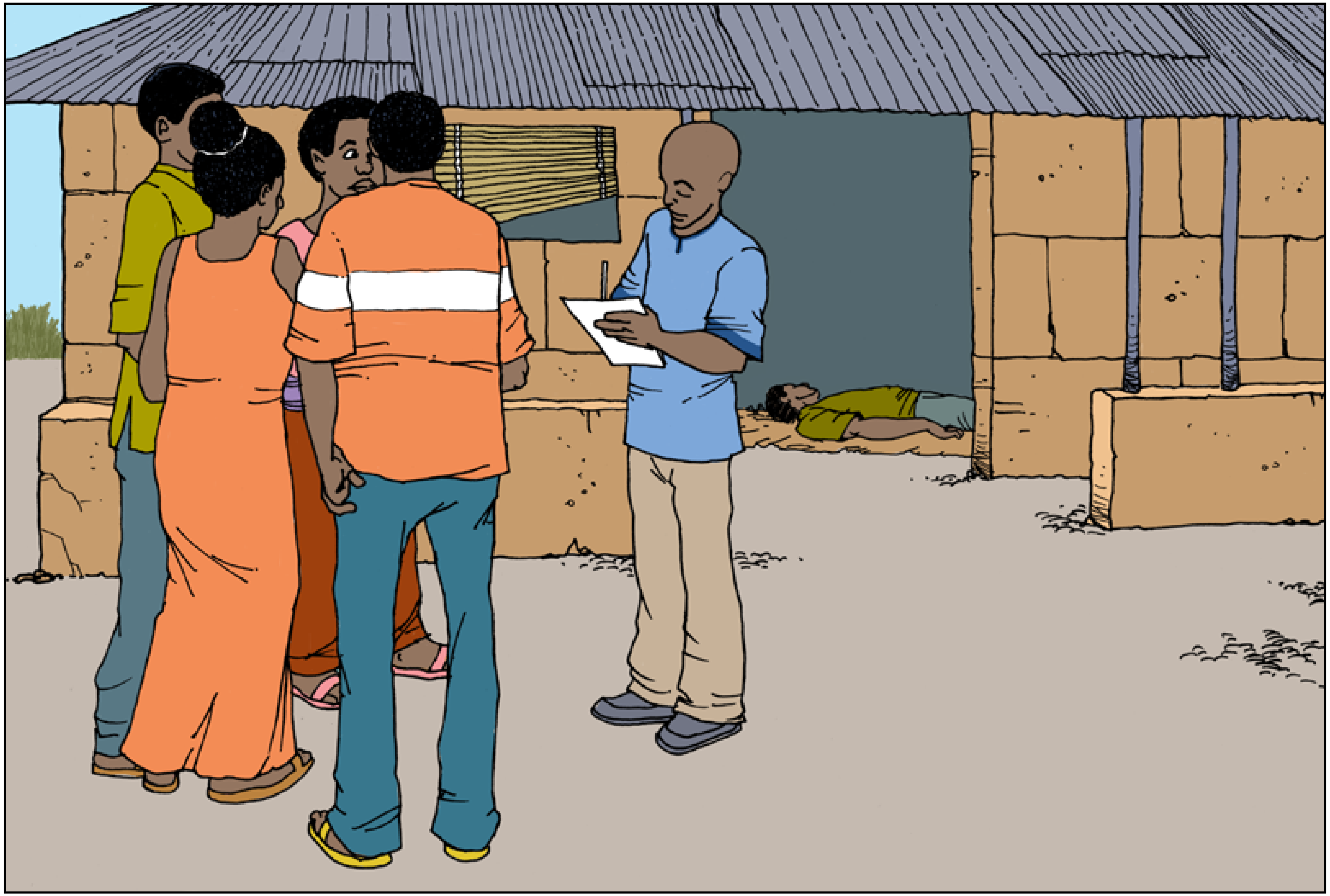

Do not touch, wash or clean any dead body. Burying all who die safely is one of the best ways to make sure we have zero cases of Ebola in Liberia. **Call 4455** to report a dead body and to alert the burial teams to pick up the body for free burial.

12/23/2014   CS252674-C

Ebola Must Go: Bury All Dead Bodies Safely-Call 4455

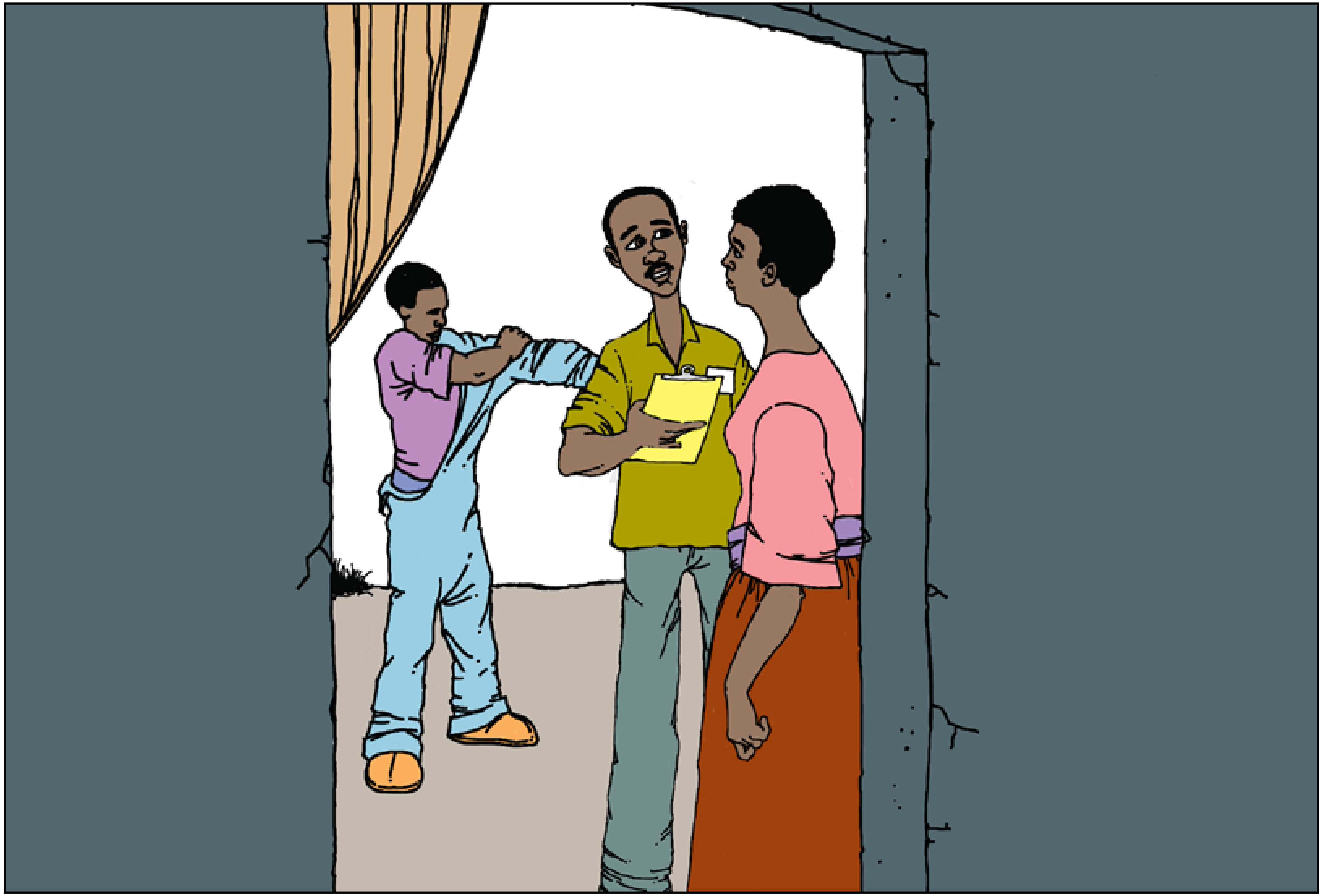

Burial teams know this kind of safe burial is very difficult for the family and the community. They will talk to the family members about the different ways they can pay respect without touching the body. All burials will be safe, free, and respect the families.

Ebola Must Go: Bury All Dead Bodies Safely-Call 4455

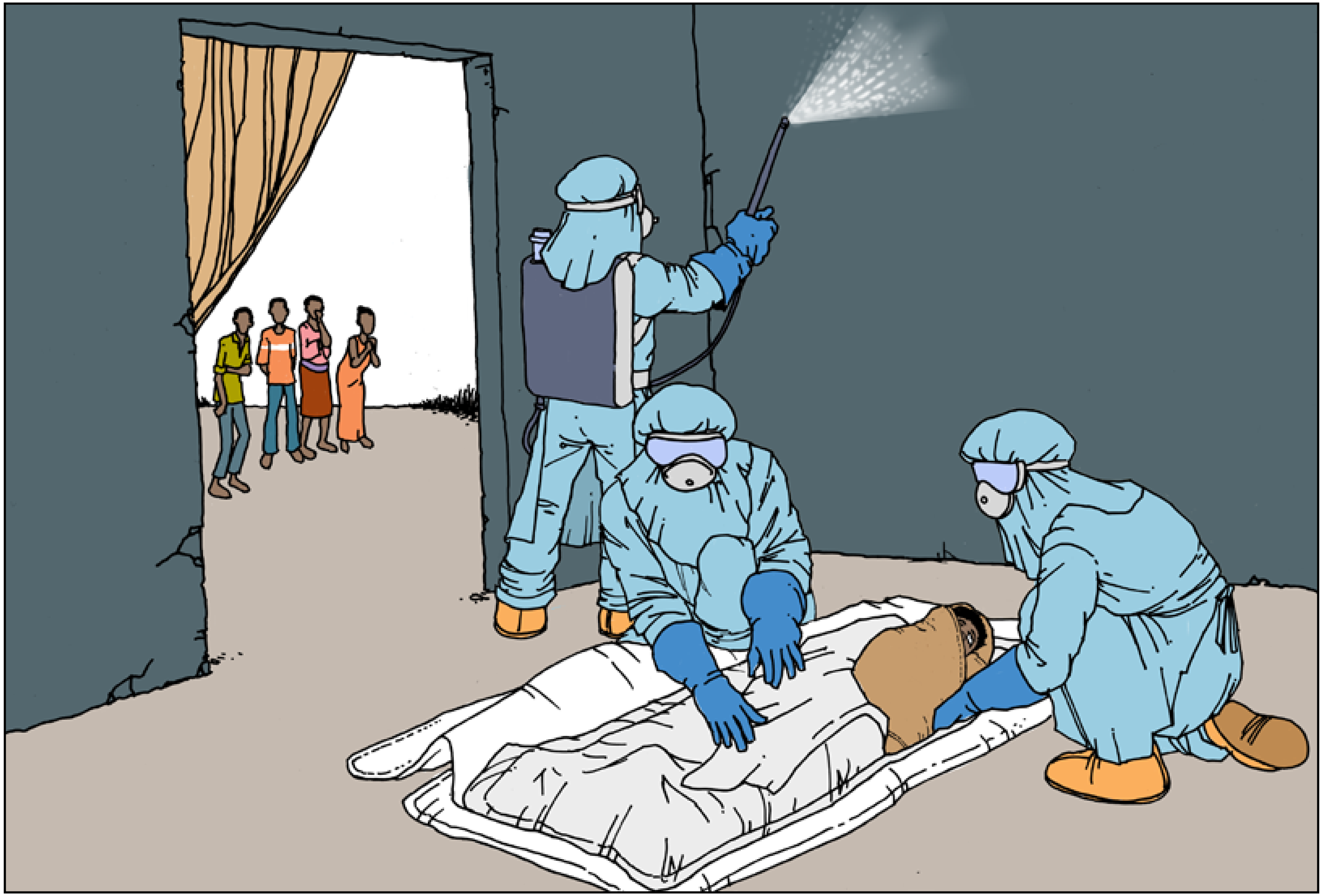

Burial teams wear special protective clothes (the overhaul suits) to keep them safe. Burial teams are watched by others and have chlorine sprayers. They spray the chlorine to clean the body and the area the body was in to kill the Ebola virus and keep the family and the community safe.

12/23/2014 CS252674-C

Ebola Must Go: Bury All Dead Bodies Safely-Call 4455

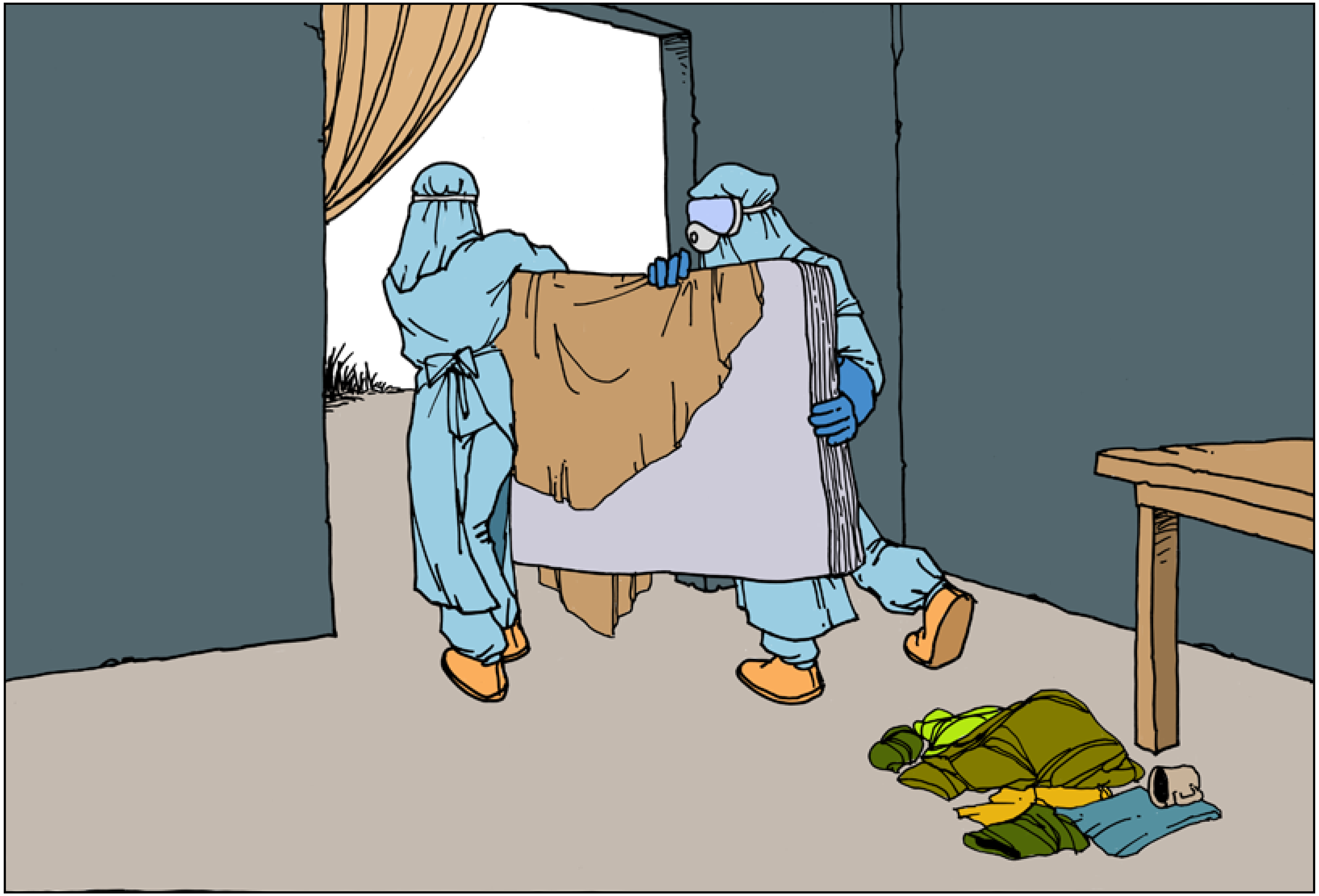

6a

The family has the right to decide if the personal things of the dead person will be burned, put in the grave with the person, or sprayed with chlorine to clean them.

Ebola Must Go: Bury All Dead Bodies Safely-Call 4455

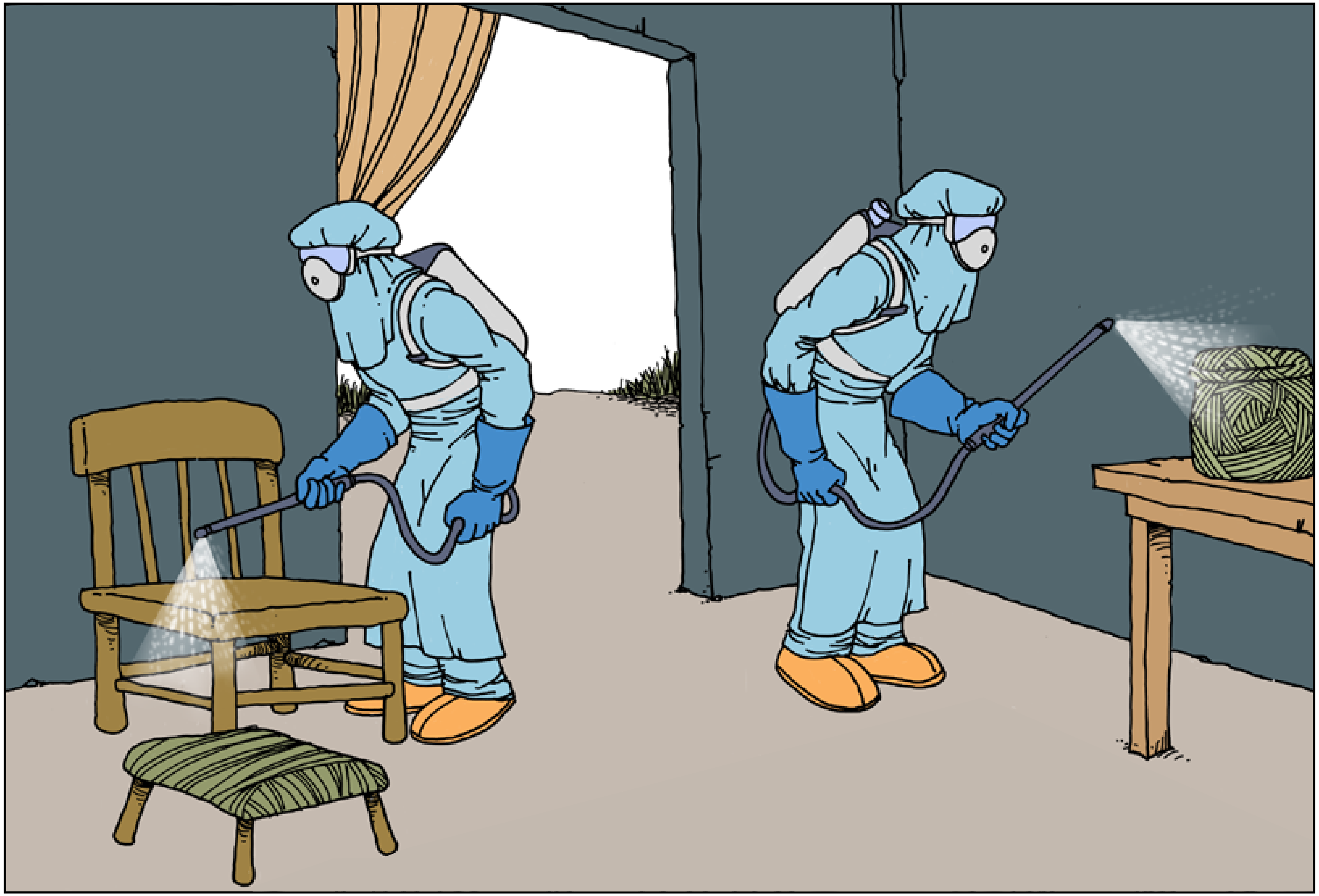

6b

Ebola Must Go: Bury All Dead Bodies Safely-Call 4455

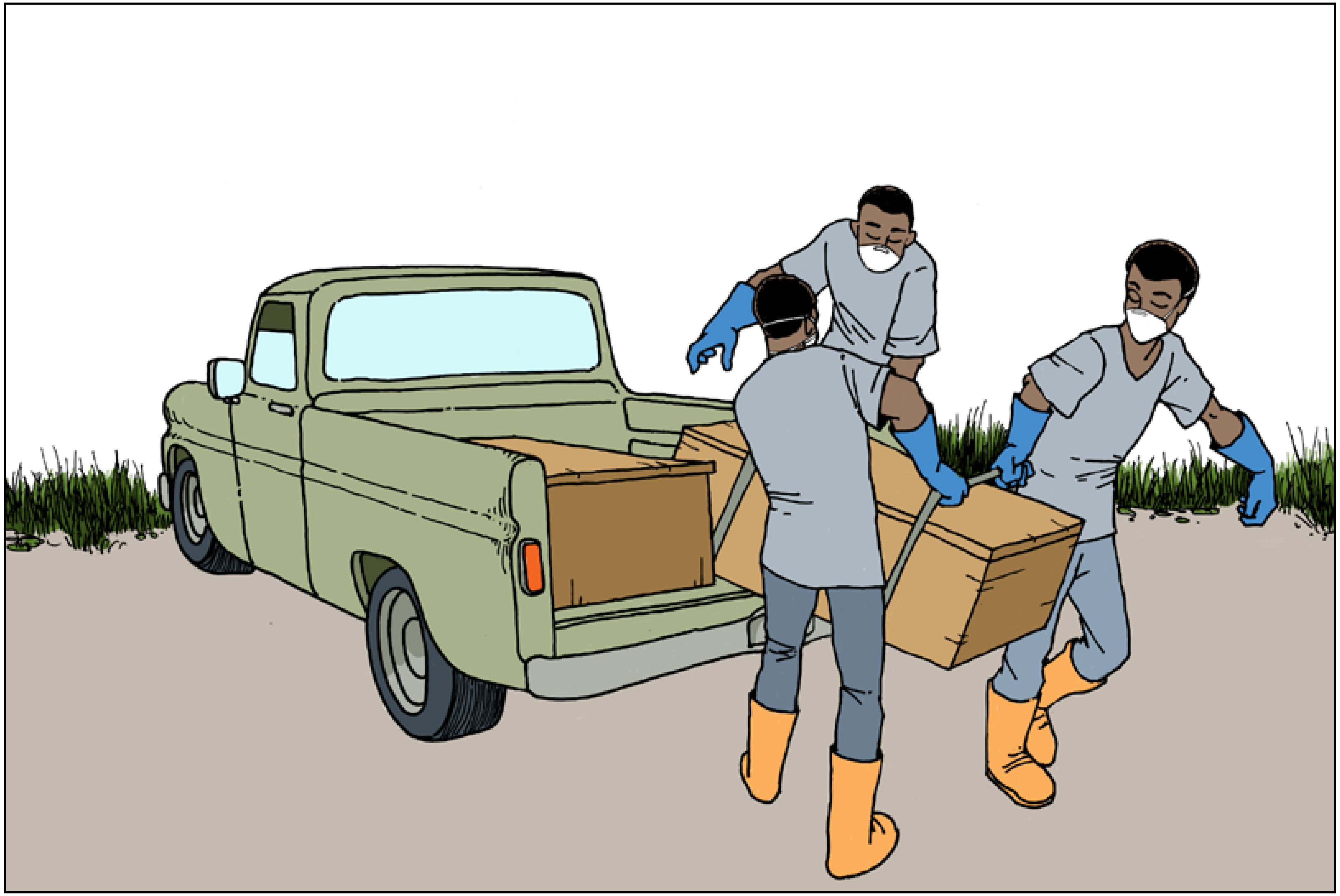

7

The burial teams will deliver the body to the cemetery. No bodies will be burned.

12/23/2014 CS252674-C

Ebola Must Go: Bury All Dead Bodies Safely-Call 4455

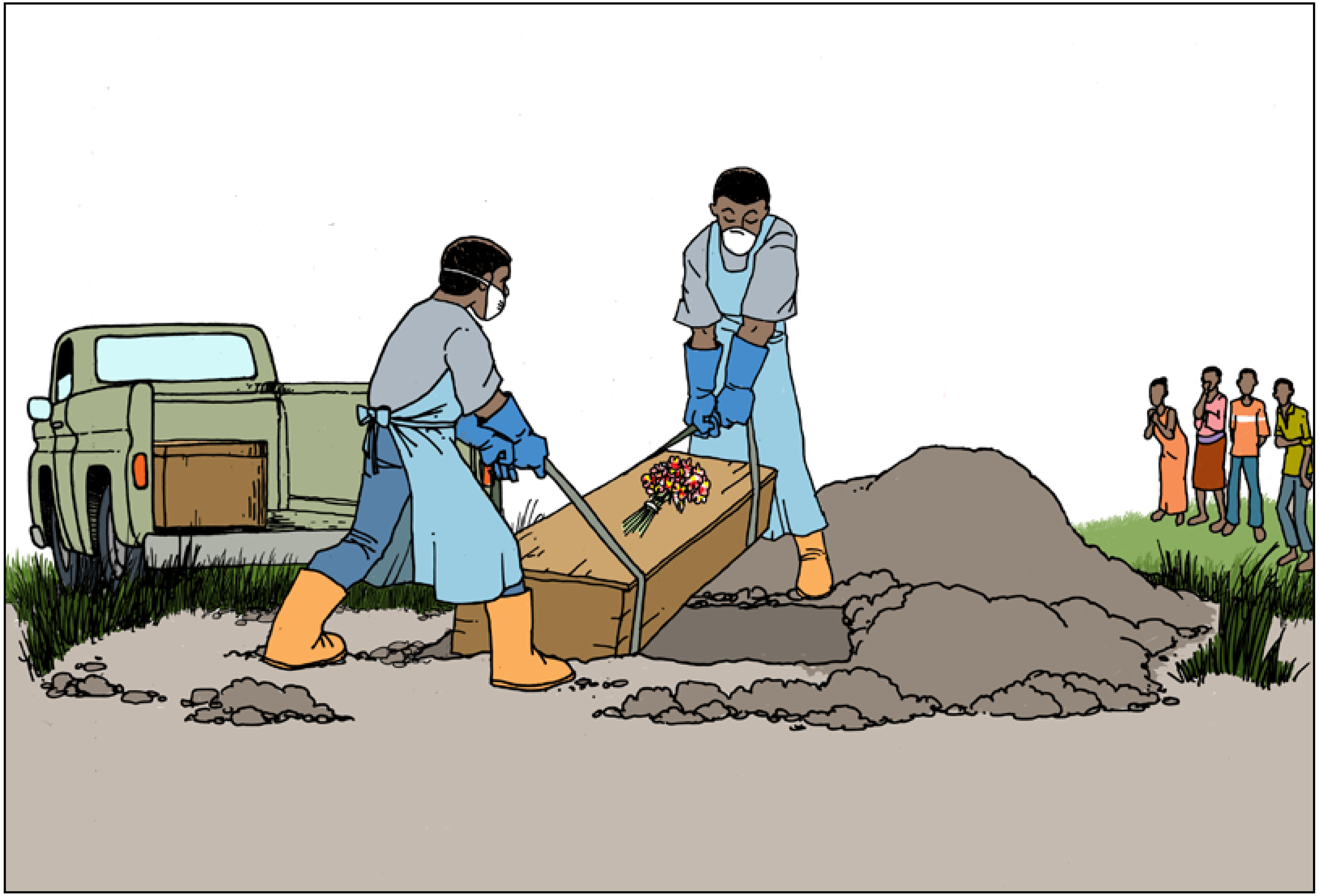

Five members of the family will be able to attend the burial. They will not travel with the burial team. The family can stand 15 feet away. A religious leader can come. The family can chose a gravestone for the family member.

12/23/2014 CS252674-C

Ebola Must Go: Bury All Dead Bodies Safely-Call 4455

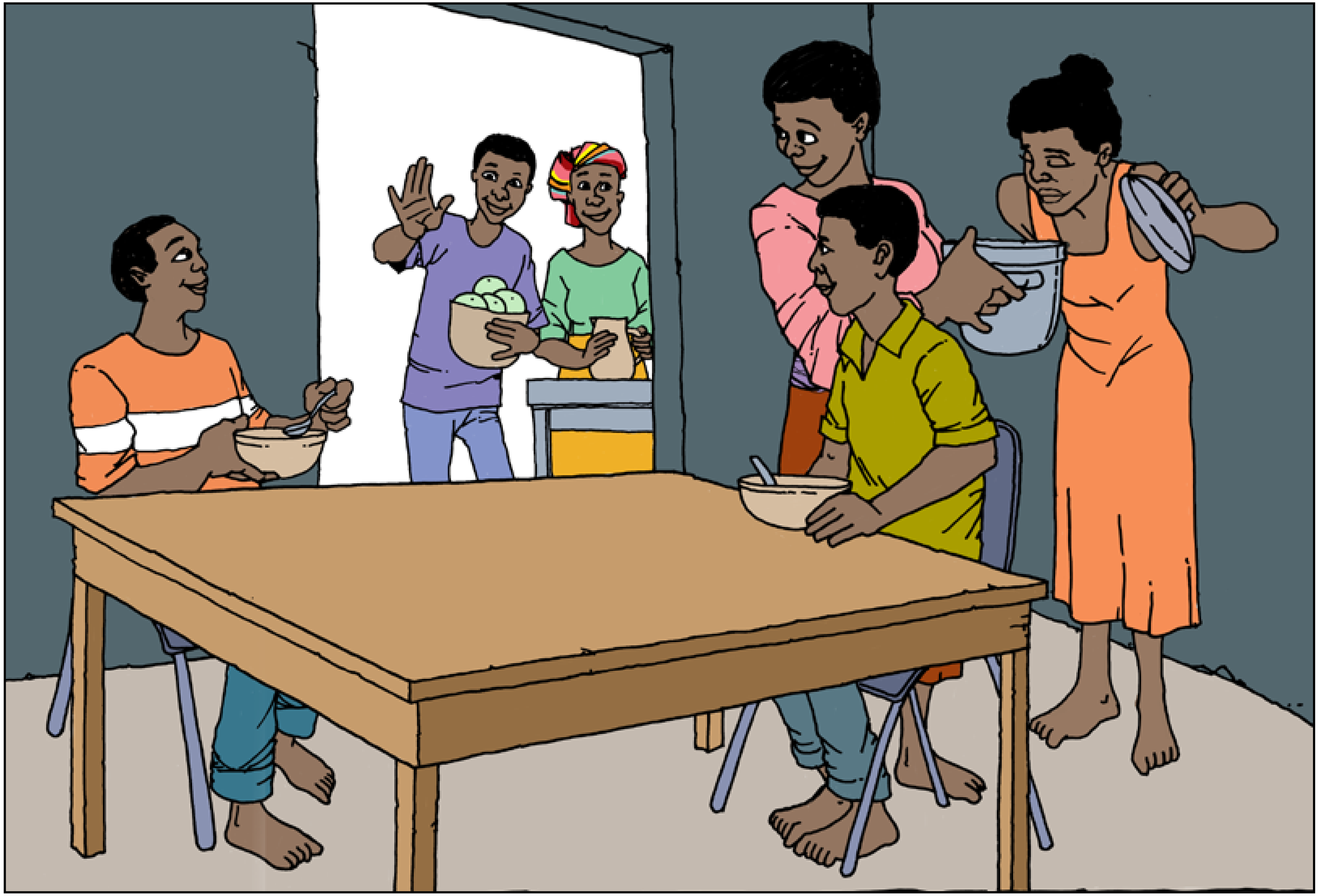

Protect yourself. Protect your family. Protect your community.

For anyone who dies, **call 4455**.

All burials will be safe, free, and respectful of the body and the grieving family.
