# Supplementary material for: Social Mobilization and Community Engagement Central to the Ebola Response in West Africa: Lessons for Future Public Health Emergencies
Source: Glob Health Sci Pract. 2016 Dec 23;4(4):626–46. doi: 10.9745/GHSP-D-16-00226 (PMC5199179; doi:10.9745/GHSP-D-16-00226)
Supplement: supplementary materials [file Supplementary_material-4.pdf]

What is contact tracing?

Contact tracing can stop an Ebola outbreak in its tracks

U.S. Department of Health and Human Services
Centers for Disease Control and Prevention

Contact tracing is finding everyone who comes in direct contact with a sick Ebola patient. Contacts are watched for signs of illness for 21 days from the last day they came in contact with the Ebola patient. If the contact develops a fever or other Ebola symptoms, they are immediately isolated, tested, provided care, and the cycle starts again—all of the new patient's contacts are found and watched for 21 days. **Even one missed contact can keep the outbreak going.**

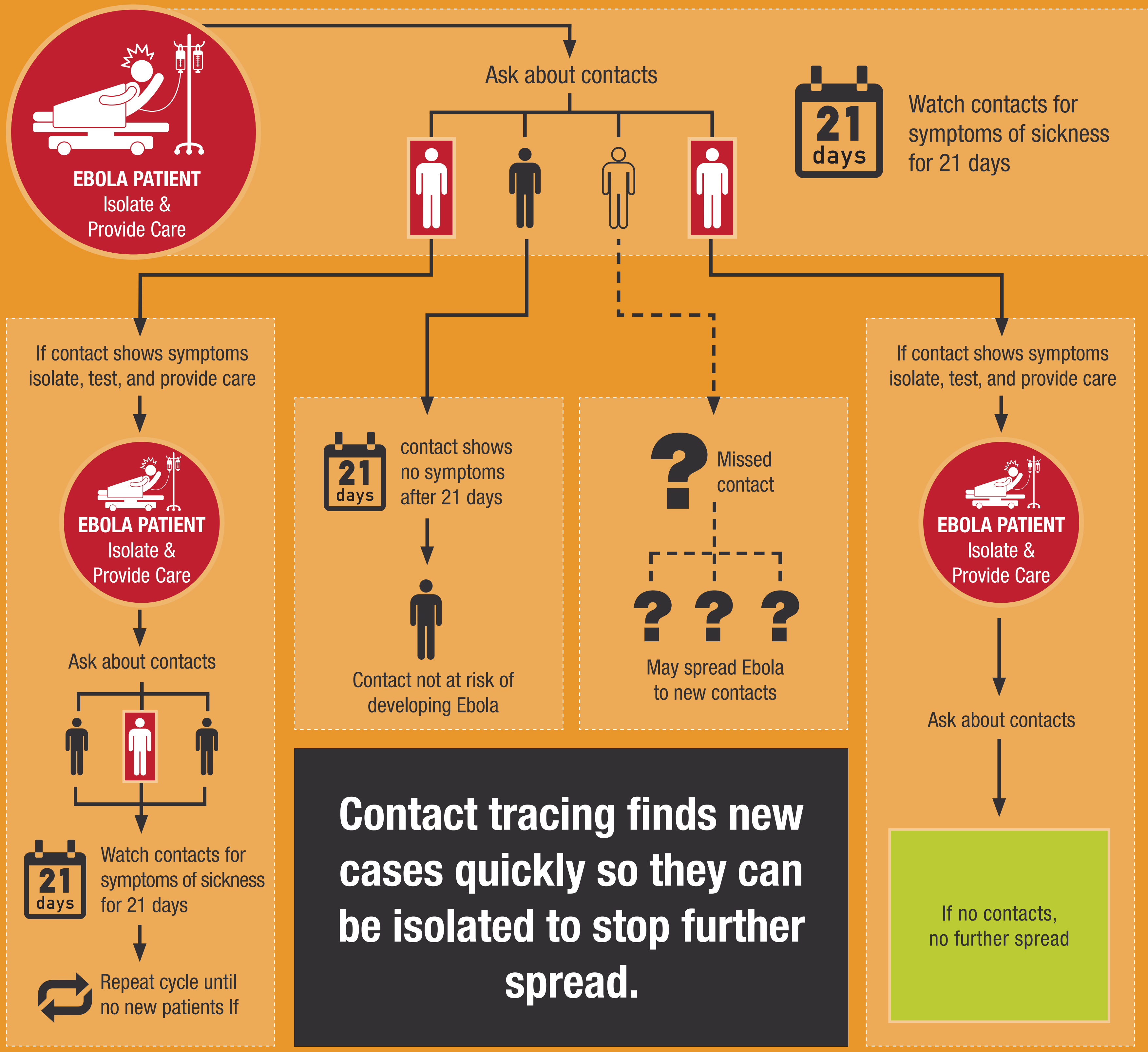

January 12, 2016 – CS262384A
