# Supplementary material for: Social Mobilization and Community Engagement Central to the Ebola Response in West Africa: Lessons for Future Public Health Emergencies
Source: Glob Health Sci Pract. 2016 Dec 23;4(4):626–46. doi: 10.9745/GHSP-D-16-00226 (PMC5199179; doi:10.9745/GHSP-D-16-00226)
Supplement: supplementary materials [file Supplementary_material-5.pdf]

# Get Early Treatment for Ebola

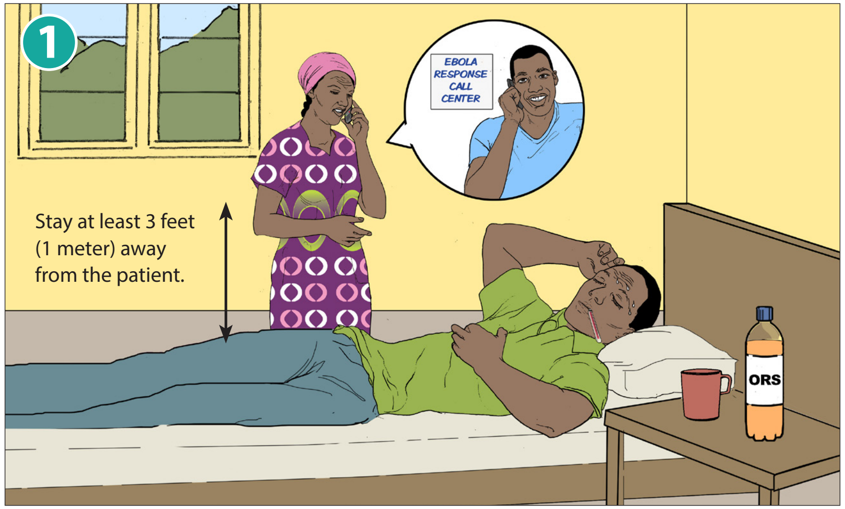


If a loved one is sick with suspected Ebola, call 117 for help. Do not touch them, their blood, or their body fluids (vomit, feces, urine, sweat). Tell them to drink plenty of Oral Rehydration Solution (ORS) or water. Patients who drink lots of ORS early have a much better chance of surviving. Do not touch the cup the patient drinks from. Refill it without touching. Wash your hands often while you wait for help.

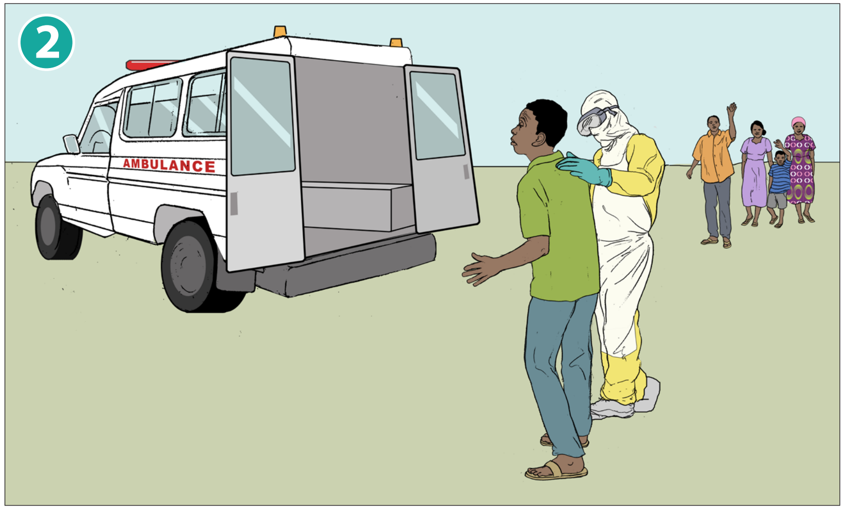


If you have a sick loved one, an ambulance is the safest way to get them to a treatment facility. Do not use taxis or other public vehicles. If your loved one is taken to a treatment facility early, they have a much better chance of surviving Ebola. This helps to protect your family too.

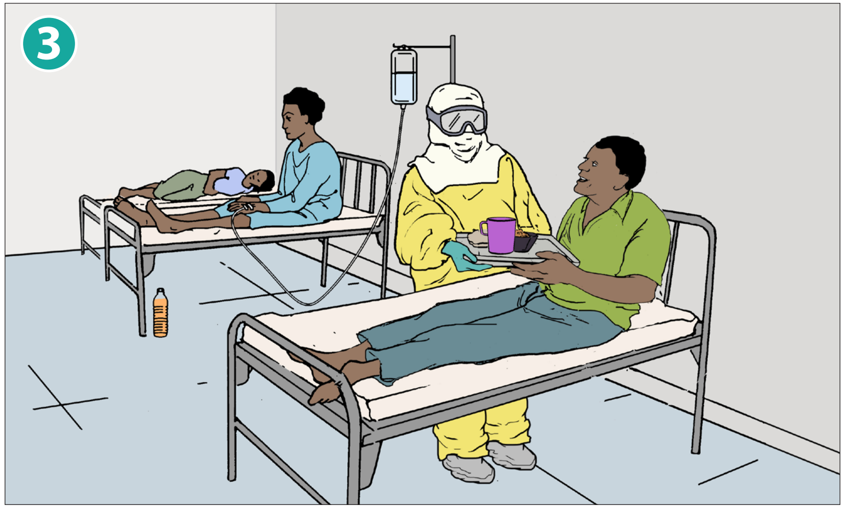


Your loved one will get the best early medical care at the treatment facility. They will get plenty of ORS and medicine for fever and other symptoms. At a treatment center, healthcare staff in protective clothing can safely care for patients, much better than at home.

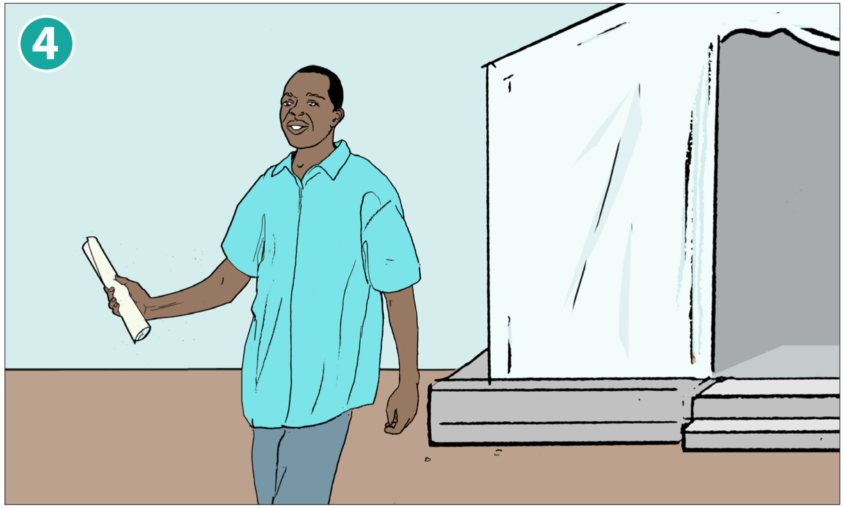


Your loved one has a much better chance of surviving Ebola if they get early medical care, soon after symptoms appear.

**U.S. Centers for Disease Control and Prevention**

CS252817-A
